# Divergent viral presentation among human tumors and adjacent normal tissues

**DOI:** 10.1038/srep28294

**Published:** 2016-06-24

**Authors:** Song Cao, Michael C. Wendl, Matthew A. Wyczalkowski, Kristine Wylie, Kai Ye, Reyka Jayasinghe, Mingchao Xie, Song Wu, Beifang Niu, Robert Grubb, Kimberly J. Johnson, Hiram Gay, Ken Chen, Janet S. Rader, John F. Dipersio, Feng Chen, Li Ding

**Affiliations:** 1 McDonnell Genome Institute, Washington University, St. Louis, Missouri 63108, USA; 2 Department of Genetics, Washington University, St. Louis, Missouri 63108, USA; 3 Department of Mathematics, Washington University, St. Louis, Missouri 63108, USA; 4 Department of Pediatrics, Washington University, St. Louis, Missouri 63108, USA; 5 Department of Medicine, Washington University, St. Louis, Missouri 63108, USA; 6 Department of Surgery, Washington University, St. Louis, Missouri 63108, USA; 7 Brown School Master of Public Health Program, Washington University in St. Louis, St. Louis, MO 63130, USA; 8 Siteman Cancer Center, Washington University, St. Louis, Missouri 63108, USA; 9 The University of Texas MD Anderson Cancer Center, Department of Bioinformatics and Computational Biology, Houston, Texas 77030, USA; 10 Department of Obstetrics and Gynecology, Medical College of Wisconsin, Milwaukee, WI 53226, USA

## Abstract

We applied a newly developed bioinformatics system called VirusScan to investigate the viral basis of 6,813 human tumors and 559 adjacent normal samples across 23 cancer types and identified 505 virus positive samples with distinctive, organ system- and cancer type-specific distributions. We found that herpes viruses (e.g., subtypes HHV4, HHV5, and HHV6) that are highly prevalent across cancers of the digestive tract showed significantly higher abundances in tumor versus adjacent normal samples, supporting their association with these cancers. We also found three HPV16-positive samples in brain lower grade glioma (LGG). Further, recurrent HBV integration at the *KMT2B* locus is present in three liver tumors, but absent in their matched adjacent normal samples, indicating that viral integration induced host driver genetic alterations are required on top of viral oncogene expression for initiation and progression of liver hepatocellular carcinoma. Notably, viral integrations were found in many genes, including novel recurrent HPV integrations at *PTPN13* in cervical cancer. Finally, we observed a set of HHV4 and HBV variants strongly associated with ethnic groups, likely due to viral sequence evolution under environmental influences. These findings provide important new insights into viral roles of tumor initiation and progression and potential new therapeutic targets.

Much of the community’s collective sequencing capacity has been devoted to cancer genomics over the last several years, with the result being that DNA and RNA data are available for thousands of tumor samples across many different cancer types. The Cancer Genome Atlas (TCGA) Pan-Cancer project has discovered numerous somatic mutations in key cancer genes^1^,^2^,^3^. For example, a recent study across 12 cancer types identified 127 significantly mutated genes involved in various cellular processes of cancer^3^. Also, mutational signatures related to endogenous and exogenous DNA damage have been found in different cancer types^4^,^5^,^6^,^7^,^8^, such as the APOBEC-associated cytosine deaminase mutational signature^4^,^5^ and smoking-related cytosine-to-adenine signatures^9^.

It is estimated that viral infection contributes to 10–15% of human cancer cases^10^. Human papillomavirus (HPV), Human hepatitis B and C (HBV and HCV), and Epstein-Barr virus (EBV or HHV4) are all well-known agents of viral-related cancers. Other viruses such as human T-cell lymphotropic virus (HTLV), Kaposi’s sarcoma virus (HHV8), Merkel cell polyomavirus (MCV), Human immunodeficiency virus-1 (HIV-1) are also associated with cancer^11^,^12^,^13^. A number of mechanisms have been described, including general disruption of human genome integrity by virus integration^14^,^15^,^16^,^17^ and virus oncogene binding to host proteins to promote tumor growth^18^. There are also epigenetic aspects of virus-host interaction, for example EBV has been implicated in the altered methylation patterns of gastric adenocarcinoma^19^ and HBV can affect the methylation of flanking human gene sequences^20^. In addition, viral microRNAs in EBV can be involved in tumor growth^21^,^22^.

The Pan-Cancer TCGA data collection is a useful, large-sample resource for studying virus-host interactions and their implications for human cancer. Early results have been obtained^23^,^24^, including HPV and HBV integration sites on host genes, but larger-scale studies of viruses in human cancers are otherwise limited. Here, we extend the field of investigation using the largest dataset to date, consisting of 6,813 tumors and 559 adjacent normal samples across 23 cancer types. Three important issues are explicitly addressed in the present study: 1) differential viral expression and integration patterns between tumors and adjacent normal samples, 2) the discovery and implications of novel rare viral insertions and 3) differences among ethnicities that may point to environmental influences on viral sequence evolution. In particular, for stomach adenocarcinoma (STAD), cervical cancer (CESC), and liver hepatocellular carcinoma (LIHC) that all have established associations with viral initiation, respective sample counts are at least three folds larger than previous studies^23^,^24^. The significantly larger collection of virus-positive samples (505 vs. 178^23^) in this study enables us to define virus frequency estimates with increased precision in various cancer types and to detect novel recurrent virus integration sites. Moreover, direct comparison to tumor-adjacent normal pairs enables detailed, unbiased formulation of portraits for different viruses, which have not yet been examined^23^,^24^. Our findings extend previous work and provide new insights to virus infection, viral gene expression, integration, and virus variants across different cancer types.

## Results

### Virus discovery across 23 cancer types

We obtained 7,372 TCGA RNA-Seq data sets from CGHub, comprising 6,813 tumors and 559 adjacent normal samples across 23 cancer types, the full names and abbreviations are listed in Materials and Methods. We developed a bioinformatics system called VirusScan (Methods, Fig. S1) for accurate identification of known viruses in these data based on the complete NCBI NT database containing all known viral sequences.

**Figure 1:**
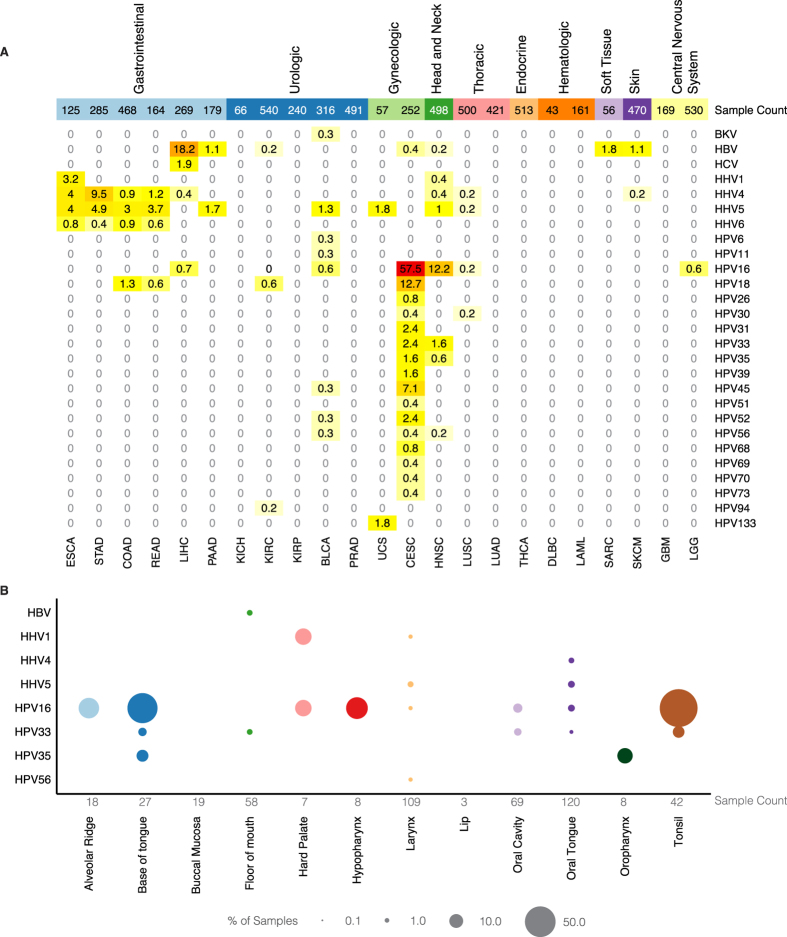
The detected frequency of various viruses across 23 cancer types, classified across (**A**) 10 different organ systems and (**B**) 12 different HNSC anatomic sites. The top number in (**A**) is the total number of samples in the given cancer type and the bottom number in (**B**) is the number of samples by anatomic site in head and neck cancers. In (**B**), circle area is proportional to frequency. The full name of each cancer type in (**A**) can be found in Materials and Methods.

We quantified virus abundance by numbers of virus-supporting reads per hundred million reads processed (RPHM). A histogram of HPV read counts shows clear bimodal distribution (Fig. S2), suggesting RPHM ≥ 100 as a reasonable identifier of virus-positive status (Materials and Methods). The RPHMs for viruses having values ≥100 in at least one sample can be found in Supplementary Data 1. We plotted the distribution of 505 virus-positive samples in each cancer type (Fig. 1A), noting that esophageal cancer (ESCA), stomach, colon, and rectal adenocarcinomas (STAD, COAD, and READ), liver hepatocellular carcinoma (LIHC), cervical carcinoma and endocervical adenocarcinoma (CESC), and head/neck squamous cell carcinoma (HNSC) all show frequent viral presence (>5%). Notably, we observed distinct virus patterns across different organ systems. For instance, high prevalence of human herpesviruses (HHVs) was found in gastrointestinal-related cancers, especially ESCA, STAD, COAD, and READ. HHV1, HHV4, and HHV5 are present in 3–4% of ESCA samples and 9.5% of STAD samples are HHV4-positive, consistent with the TCGA gastric adenocarcinoma report^19^ (Figs S3A and S4). HHV4 is often present at RPHM > 10^4^ and two STAD samples and one COAD sample are positive for both HHV4 and HHV5. Conversely, HBV and HCV are prevalent in LIHC (18.2% and 1.9%, respectively) (Fig. S3A).

We found that 14.6% of HNSC samples are HPV-positive, with the majority subtype being HPV16. The latter figure is comparable to previous observations^25^ and consistent with studies showing positive associations between HPVs, especially HPV16, and HNSC^25^,^26^. Strains show variable site preferences in HNSC (Fig. 1B); HPV16 and HPV33 are most prevalent in the tonsil (76.2% and 7.1% of all tumor cases, respectively) and the base of tongue (48.1% and 3.7%, respectively), while HPV35 is more common in the oropharynx and the base of tongue (12.5% and 7.4%, respectively). The body of findings is considerably larger (supplementary information) and comprises some notable first observations. For example, we discovered three HPV16-positive samples in 530 brain lower grade glioma (LGG) samples. There has been controversy over HPV16 infections in the central nervous systems (CNS)^27^,^28^. We reported a low detected frequency of HPV16 in LGG samples.

A virus signature is often observed in the form of a single dominant virus subtype in each individual tumor (Fig. S3A,B). This may reflect an evolutionary victory over other subtypes during tumor progression or the specific adaptation of a virus to the particular cell type. However, we did observe some exceptional cases that were positive for two different viruses, for example, one STAD case having HHV4 and HHV5, one LIHC case having HBV and HPV16 (Fig. S3A) and one BLCA with having HPV6 and HPV11 (Fig. S3C). Since we only show the viruses with RPHM > 100, we did not rule out the possibility of the co-infection with other low-abundance viral species (RPHM < 100)^29^.

### Comparison of viral abundance in tumor and adjacent normal pairs

**Figure 2:**
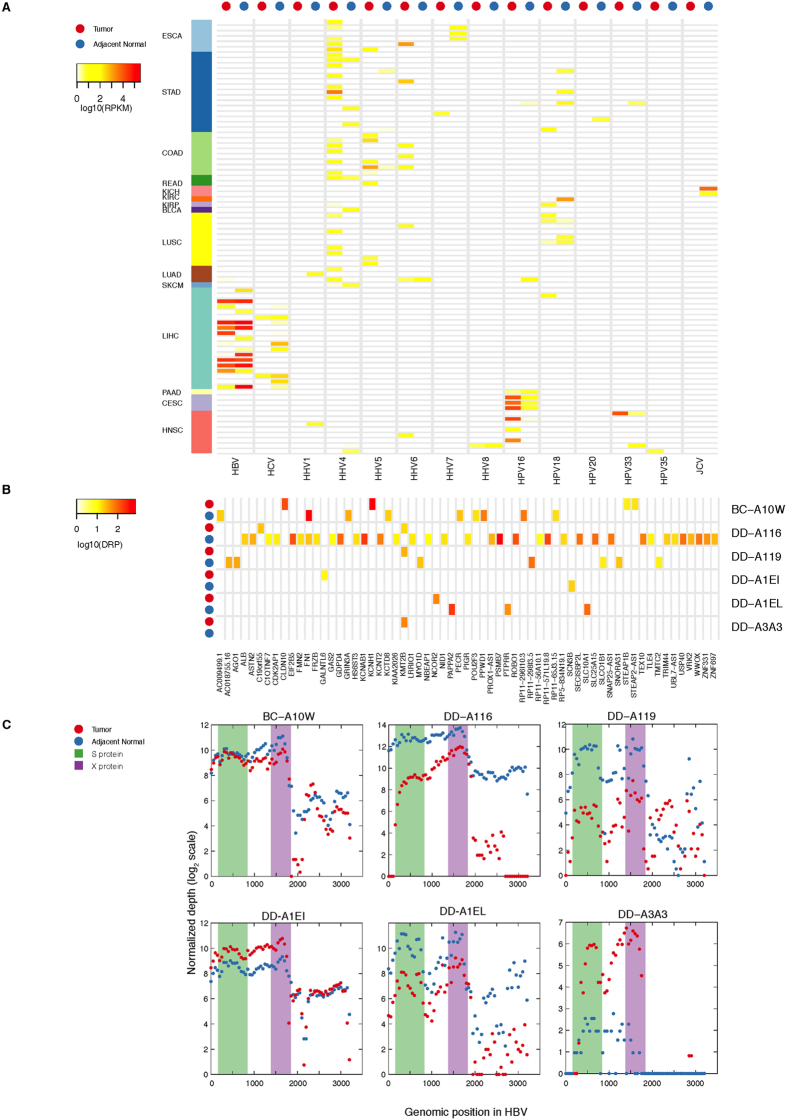
(**A**) Comparison of virus abundance in 81 tumor-adjacent normal pairs across 15 cancer types, with inclusion based on either the tumor or normal tissue showing a virus signature (RPHM ≥ 5). Red and blue denote tumor and normal samples, respectively, with cancer types represented by the standard color palette. Virus abundance is quantified by a white (zero) to red (maximum) continuum. (**B**) Comparison of HBV integration sites for six tumor-adjacent normal pairs. In six tumor/adjacent normal pairs, both tumor and normal samples have HBV abundance RPHM ≥ 100. The color bar shows the number of discordant read pairs (DRP) in log10 scale. Two recurrent sites, *FN1* and *KMT2B*, are found in adjacent normal and tumor samples, respectively. (**C**) The comparison of HBV gene expression in 6 tumor-normal pairs. For a direct comparison, we use the normalized depth (see Methods) to quantify the gene expression.

Among the 559 adjacent normal samples with available RNA-Seq data, we found 81 tumor-adjacent normal pairs across 15 cancer types that showed varied virus signatures for HBV, HPV and HHV. Chronic infection with HBV or HCV is a known risk factor for liver cancer^30^,^31^. For HBV, the abundance in adjacent normal sample is at least comparable, but often appreciably higher than in the paired tumor (Fig. 2A). There were two extreme cases (TCGA IDs: DD-A11A and DD-A1EH), one with tumor RPHM > 10^4^ but complete viral absence in the adjacent normal sample and the other with adjacent normal RPHM > 10^4^ and very low viral presence in the tumor. The clinical-pathological information shows that patient DD-A1EH with only HBV in adjacent normal has a family history of cancer, and sample DD-A11A with HBV in tumor has no family history of cancer, which may indicate different cancer etiologies, i.e., inherited mutations and HBV infection, respectively. We also looked the expression data of 624 cancer genes for the two tumors^32^. We use TCGA firehose RSEM data to quantify the gene expression. The mean RSEM values (log2 scale) of DD-A11A and DD-A1EH are 8.09 and 8.4, respectively. The p-value of the difference from paired t-test is 0.0001. For HCV, we found viral abundance to be higher in adjacent normal samples than tumors in most cases (p-value = 0.02, paired t-test). The lack of positive correlation between viral abundance and liver cancer is unexpected. Expression of HBV and HCV genes may be needed to sustain chronic infection, but are perhaps not sufficient for initiation and/or progression of the cancer. Lower expression of the virus in the tumor may also be related to evading host immune system response. We show below that the integration patterns of HBV are strikingly different between tumor and adjacent normal sample, which may be a key factor for liver tumorigenesis. No integration sites were found for HCV in our study, consistent with the literature^33^. In contrast, the viral abundance of HPV16, the leading HPV subtype associated with both HNSC and CESC^26^,^34^, is significantly higher in tumor than the paired adjacent normal sample (p-value = 0.02; Fig. 2A). HHV4, HHV5, and HHV6 tend to be found in gastrointestinal cancers and are also absent in paired adjacent normal samples (Fig. 2A), with p-values as <0.001, <0.001 and 0.005, respectively. Previous studies demonstrated that HHV4 is associated with gastric adenocarcinoma^35^. Our observations suggest that HHV5 may also play pivotal roles in the tumorigenesis of organs within the gastrointestinal system. In addition, in many tumor-adjacent normal pairs, we observed HHV1, HPV20, HHV7 and JCV in adjacent normal samples, but absent in the tumors, which suggest they are part of the human flora.

### Viral infection associated expression profiles in tumors and adjacent normals

We compared gene expression profiles for the tumor-adjacent normal pairs, where both tumor and matched adjacent normal were found to be HBV-positive (RPHM ≥ 100). This criterion returns 6 tumor-adjacent normal pairs (Fig. 2B,C).

Overall, the HBV X protein is the highest expressed gene in both tumors and adjacent normal pairs (Fig. 2C), followed by the S protein. No significant difference was observed in terms of the expression profile between tumors and their matched normal samples. The HBV X protein plays an important role in virus replication, the pathogenesis of chronic liver disease^36^, and the development of hepatocellular carcinoma^37^,^38^. Comparison of HBV’s virus integration sites for tumor-adjacent normal pairs showed that virus integration sites in tumors were not detected in the matched adjacent normal samples (Fig. 2B). Importantly, in these six tumors, we found three samples with HBV virus integration in *KMT2B* (*MLL4*), suggesting the integration sites in this gene are important for the development of liver cancer, consistent with other studies^16^,^23^. We note sample DD-A116 has a very large number of HBV integration sites compared to other samples. Some of integration sites may be false positive from the artificial discordant read pairs. Direct comparison of six tumor-adjacent normal pairs here shows that different virus integration sites are present in tumor and adjacent normal cells, suggesting that the specific virus integration patterns may be a key factor for driving liver tumorigenesis.

**Figure 3:**
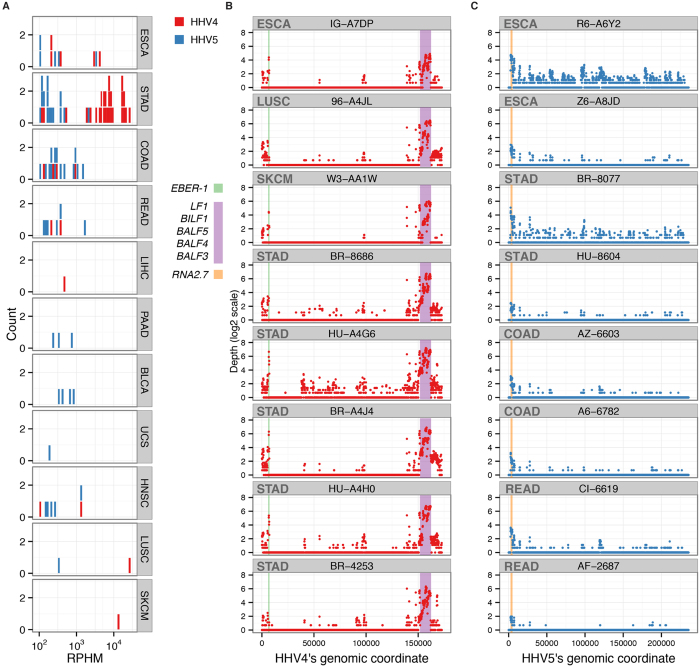
(**A**) The histogram of HHV4 and HHV5-postive samples across different cancer types. Comparison of depths for two different viruses: (**B**) HHV4, and (**C**) HHV5, across the entire virus genome. X-axis is genomic position and y-axis is the calculated sequencing depth.

We found that a total of 43 samples, including five ESCA, two HNSC, 27 STAD, four COAD, two READ samples, one LIHC, one LUSC and one SKCM samples, are HHV4-positive (RPHM ≥ 100) (Fig. 3A). Figure 3B shows expression profiles for eight selected samples in four different cancer types and how they share similar expression patterns. There are two particular regions where expression seems to dominate: the small RNA *EBER-1* gene and a second region that includes genes *LF1*, *BIF1*, *BALF3*, *BALF4*, and *BALF5*. *EBER-1* has been shown to bind the dsRNA-activated inhibitor of the DNA-dependent activator of IFN-regulatory factors (DAI)^39^. DAI is a protein kinase that specifically phosphorylates polypeptide chain initiation factor elF-2. There is also some evidence for an association between *EBER-1* and tumor morphology and primary site^40^. The second region contains three glycoproteins BALF3, BALF4, and BALF5. BALF4 can dramatically enhance the ability of HHV4 to infect human cells^41^. The detection of highly abundant HHV4 in ESCA, LUSC, and SKCM and similar expression profiles to STAD suggest its tumorigenesis in these three cancer types. For the majority of other samples having HHV4’s RPHM values ≥100, we also observed the expression of the two regions (Fig. S5). For instance, we found that *EBER-1* is highly expressed in BR-8676. We also detected high expression of *LF1*, *BIF1*, *BALF5*, *BALF4* and *BALF3* in samples L5-A34H and LN-A49S.

Our analysis detected 53 HHV5-positive samples across nine cancer types, comprised of five ESCA, 14 STAD, 14 COAD, six READ, three pancreatic adenocarcinoma (PAAD), four bladder urothelial carcinoma (BLCA), one uterine carcinosarcoma (UCS), five HNSC and one lung squamous cell carcinoma (LUSC) samples (Fig. 3A), consistent with a previous report^42^. Figure 3C shows the gene expression of HHV5 in eight representative samples. *RNA2.7*, a 2.7-kb RNA gene that inhibits apoptosis^43^, shows the highest expression across samples. Because disruption of apoptosis can lead to tumor initiation, progression, or metastasis^44^, *RNA2.7* can be regarded as a viral oncogene. It was also expressed in other HHV5-positive samples (Fig. S6). For HHV1 and HHV6, we did not observe differential expression profiles comparable to HHV4 and HHV5 (Fig. S7A,B).

Gene expression patterns for HPV16 and HPV18 (Fig. S8A,B) in HNSC and CESC are largely consistent with the findings in previous reports^23^,^45^,^46^. Viral genes *E6* and *E7* were expressed in most samples, while *E2*, *E4*, and *E5* were expressed in fewer samples, and *L1* and *L2* were expressed only occasionally. HPV16 and HPV18 share similar expression profiles. For three HPV16-positive LGG samples, *E6* and *E7* were expressed, but *E5*, *L1* and *L2* were not (Fig. S9). *E6/E7* were involved in binding and degrading p53/Rb proteins^47^ and their expression suggests that HPV16 may also play a role in the tumorgenesis in these LGG samples.

### Recurrent viral integrations and their effect on exon-level expression

**Figure 4:**
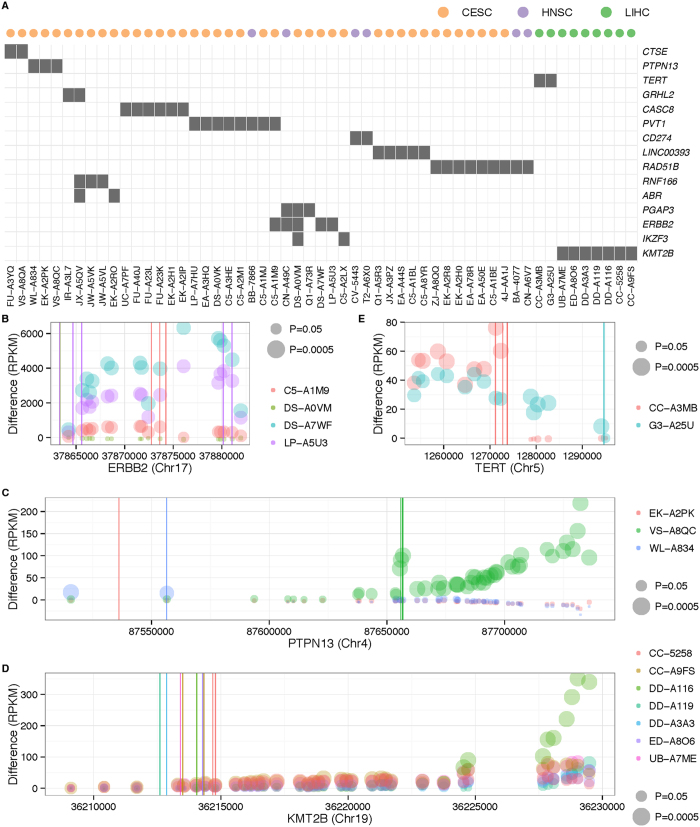
(**A**) Genes with recurrent virus integrations in LIHC (Green), HNSC (Purple), and CESC (Orange). The gray box indicates sample (x-axis) with virus integration in the specific gene (y-axis). (**B**) Differences in RPKM between case and the mean value of controls (without viral infection) for various exons in the longest transcripts of four important genes (*ERBB2, PTPN13*, *KMT2B*, *TERT*) with recurrent virus integrations. Circle area is proportional to −log10 of the difference p-value. The x coordinate of the circle’s center represents the mid-point of each exon, which is ordered from the left to right for positive strand gene and the right to left for negative strand gene. *PTPN13*, *KMT2B* and *ERBB2* are positive strand genes and *TERT* is a negative strand gene. Different samples are marked by different colors.

Discordant read pair analysis using Pindel^48^ (see Methods) revealed a battery of genes having recurrent HPV integrations in several cancers (Fig. 4A). The discordant read pairs from Pindel can be found in Supplementary Data 2 and 3 for CESC and HNSC. There are four hotspots for CESC: 1) Genes *GPHL2*, *CASC8*, *CASC11* and *PVT1* (sixteen samples), 2) *RAD51B* (eight samples), 3) *PGAP3*, *ERBB2*, and *IKZF3* (six samples), and 4) *LINC00393* (five samples). We show the location of these common integration sites on Table S2. The first hotpot is close to the *MYC* region in chromosome 8q24.21, which is a well-known HPV integration site^23^,^49^. *CASC8* and *PVT1* are two genes nearest to *MYC* with recurrent HPV integration sites. We compared the *MYC* expression for samples with HPV integrations on *CASC8* and *PVT1* and samples without these integration sites; see Fig. S10. We found that the samples with *PVT1* have a significantly higher *MYC* expression (P = 0.0089). *MYC* is an oncogene contributing to the development of many human cancers^50^, but our finding of HPV integration sites such as *PVT1* near *MYC* particularly increases its expression in genital cancers and provides one mechanistic aspect of how HPV integrations cause cancer. In addition, the current study shows that HPV16 (8 samples), HPV18 (6 samples) and HPV45 (2 samples) can all integrate into this region. The second hotpot of recurrent virus integration is at the *RAD51B* locus in 8 CESC samples, which include 3 samples with HPV16, 2 samples with HPV39, one with HPV18 and one with HPV45; interestingly, two HNSC tumors also harbor HPV16 integration at *RAD51B*. Whole-genome sequencing analysis reveals that HPV integration amplifies the somatic copy number of this region^17^. The third hotspot is centered at *ERBB2* on chromosome 17q12. The integration sites come from HPV16, which is again consistent with previous findings^23^. Finally, the fourth hotpot is recurrent integration at lncRNA *LINC00393*, including HPV16 (two samples), HPV18 (one sample), and HPV45 (two samples). Recurrent HPV integrations at *RAD51B*, *ERBB2* and *LINC00393* have also been previously described^23^. Three samples (C5-A1M9, DS-A7WF, LP-A5U3) with viral integration at *ERBB2* locus showed significantly increased expression across all exons (P-value < 0.05) (Fig. 4B). In addition, we found two samples with virus integration at *CTSE* and one sample with integration at *GPHL2* showing higher expression across the exons (Fig. S11A). On the other hand, for *RAD51B*, we did not observe a consistent increase of exon-level expression in samples with virus integration (Fig. S11A). The expression of other recurrent genes is shown in Fig. S11A,B.

We also found new recurrent integration sites at *PTPN13* in three CESC samples (Fig. 4A). *PTPN13* is a tyrosine phosphatase (PTP) enzyme that is involved in control of cell growth, proliferation, differentiation and transformation^51^. A previous study suggested that the binding motif of HPV induces *PTPN13* loss^52^ and we found two samples with HPV16 and one with HPV18 integration sites at *PTPN13*. In order to study the post-transcriptional effect of virus integration, we calculated reads mapped per kilobase per million mapped reads (RPKM) for the exons in *PTPN13* and compared the exon-level expression between integration positive cases and the mean of samples without virus integration. As shown in Fig. 4C, HPV18 integrates at intron 1 of *PTPN13* in sample EK-A2PK, while HPV16 integrates at exons 2 and 14 for samples WL-A834 and VS-A8QC, respectively. The virus genes involved in these integration sites are *E1, E4, E5, E6* and *E7*; see Table S6. Sample WL-A834, which has virus integration at exon 2, has a high expression of exon 2 and nearby exon 1. Sample VS-A8QC, which has virus integration at exon 14, has likewise high expression of nearby exons. The distinct virus integration sites and the consistent increase of the expression of the integrated or nearby exons provide strong evidence of novel recurrent HPV integrations within *PTPN13*.

In LIHC, we found two genes (*TERT*, and *KMT2B* (a.k.a. *MLL4*)) with recurrent HBV integration. *TERT* and *KMT2B* are already known in this context from whole-genome sequencing^16^. All discordant read pairs from Pindel^48^ can be found in Supplementary Data 4. Table S2 shows the genomic location of HBV integration sites on *TERT* and *KMT2B*. For example, *TERT* was recently implicated by somatic events or viral integration in hepatocarcinogensis^53^. Figure 4D and Table S2 show that the integration sites on *KMT2B* are between exon 3 and exon 8 and integrations often lead to increased expression of exons following these sites; this holds true for *TERT*, as well (Fig. 4E). For instance, the HBV integration sites in sample G3-A25U were found in the region between exons 1 and 2. In sample CC-A3MB, the HBV integration sites are between exon 6 and exon 8; see Table S6. These similar integration sites were also reported in ref. 24. Our study shows that the exons’ expression increases spontaneously after the integration sites, demonstrating the post-transcriptional effect of HBV integrations on TERT expression.

### Virus variants and their association with clinical features

We examined virus variants for HHV4, HBV, and HPV16 across samples. Variants were called by SAMtools^54^ on samples having both RPHM ≥ 1000 and sites with coverage >10X. Here we increase the RPHM cut-off from 100 to 1000 for variant analysis since we cannot obtain any HBV variant sites with coverage >10X across all selected samples using the lower cut-off. Figure S12A,B show the number of virus variants and the mutation rate for these viruses across different samples. The average number of variants for HHV4, HBV and HPV16 are 121, 52 and 22, respectively. The mutation rates for HHV4, HBV and HPV16 are 5, 49 and 4 per Kb of the reference genome, respectively. Using RPHM ≥ 1000 and sites with coverage >10X, we selected 50 variant sites for HBV across 50 HBV-positive LIHC samples, 101 variants across 24 HHV4-positive STAD samples, 17 variants across 60 HPV16-postive HNSC samples and 22 variants across 142 HPV16-positive CESC samples. With a sufficient number of samples and variants for these viruses, we next performed the clustering analysis between virus variants and ethnicity groups.

**Figure 5:**
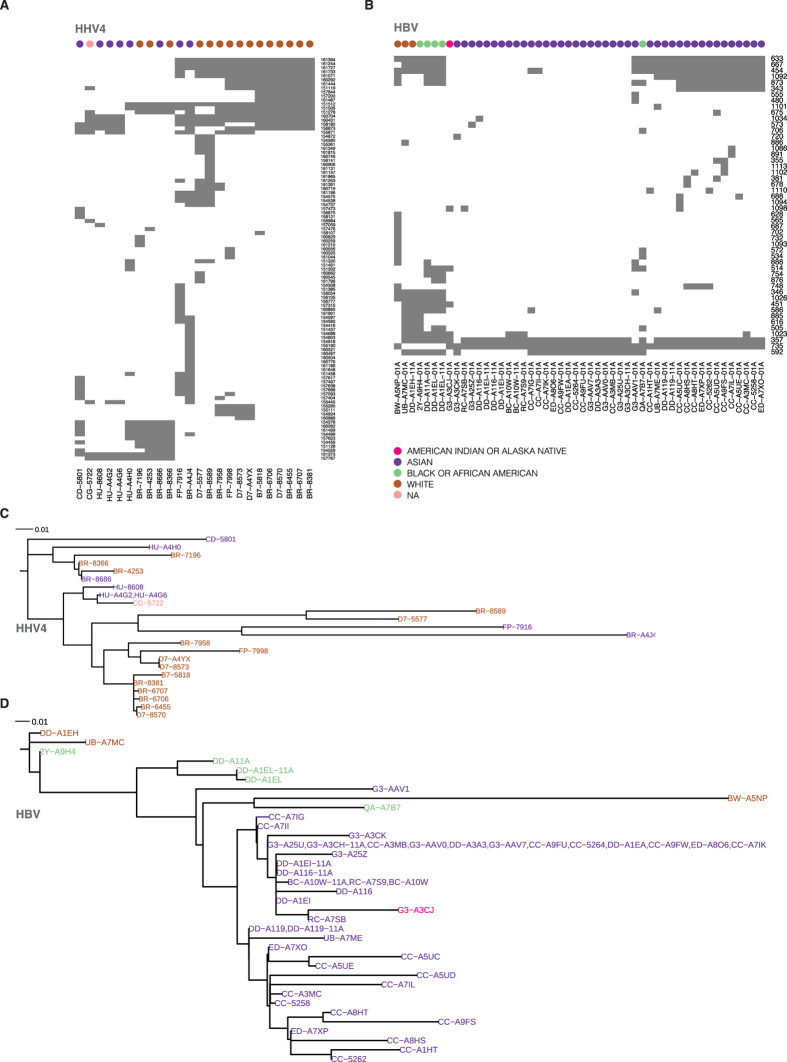
Unsupervised clustering (**A,B**) and phylogenetic analyses (**C,D**) based on HHV4 variants and HBV variants from PHYLIP. All variants have >10X coverage across all samples. In (**A**,**B**), the x-axis and y-axis are sample ID and virus genomic coordinates, respectively. The suffix “11A” in the sample IDs is an abbreviation of adjacent normal samples following TCGA convention.

Figure 5A shows the unsupervised clustering results for HHV4 variants across HHV4-positive samples with Caucasian and Asian cohorts separated in distinct groups. A separate Asian cluster signature was also observed for HBV (Fig. 5B), suggesting the association between virus genotype and ethnic group. Phylogenetic analyses gave similar results (Fig. 5C,D). For instance, for HBV, the Asian cohorts can be separated to two distinct groups based on the presence or absence of six variants located at bps 343, 454, 633, 667, 873 and 1092. Three variants, C → T at sites 343 and 454 and G → A at site 633, result in amino acid substitutions, L418F and P455S in HBV polymerase protein and R160K in S protein, respectively. HBV also acquires new variants outside the Asian cohort, such as bps 346, 451, 505, 586, 616, 885, 1023 and 1026 (Fig. 5B). Variants C → T at sites 505 and 586 and A → G at site 616 are missense mutations, which lead to the respective amino acid substitutions H472Y, R499W and I509 V in HBV polymerase. Finally, the tumor and adjacent normal pairs have the same variants for the sites observed in Fig. 5B, except for sample DD-A116, in which HBV found in the tumor has an additional variant (A → G) at site 1034, leading to an amino acid substitution Q648R in the HBV polymerase. These observations support previous studies^55^,^56^ suggesting the coevolution between HBV and the host.

We also examined viral variation by cancer type by comparing HPV16 variants between CESC and HNSC samples. These are fairly large groups having 61 HPV16-positive HNSC and 145 HPV-positive CESC samples. Figure S13 shows the frequency distributions of their variants. Most HPV16 variants overlap between the two cancer types, which suggests they reflect population diversity rather than tissue origin. We clustered these variant sites using the inclusion rules stated above for HBV and HHV4 analysis, with results shown in Fig. S14A,B, respectively. We did not observe any strong correlation between HPV16 variants and ethnic group.

## Discussion

We performed the largest investigation to date of the viral basis of human tumors across 23 cancer types using the VirusScan pipeline developed in house. In addition to the expected high prevalence of HPVs in HNSC and CESC, we also identified HPV-positive samples in other cancer types, including BLCA, brain lower grade glioma (LGG), kidney renal clear cell carcinoma (KIRC), COAD, READ, and LIHC. HPV subtypes in BLCA are HPV45, HPV51, HPV56 and HPV6 and virus abundance in four samples is especially high (RPHM > 10^4^). However, abundance in LGG, KIRC, COAD, READ, and LIHC is relatively low (between 10^2^ and 10^3^). We also observed instances having RPHM < 100 (Fig. S2), but these observations are likely to be virus-negative samples affected by contamination or cross-mapping. HPV16 predominates in LGG and LIHC, while HPV18 prevails in COAD, READ, and KIRC. This contrasts with previously low observations of HPV18 abundance in these cancer types^57^, which may have been affected by the contamination of HeLa cells^57^. No HPV infection was detected in READ, in contrast to squamous cell carcinoma of the rectum that showed a clear HPV association^58^. This observation demonstrates a different viral etiology for the two cell types. Virus-host fusion analysis identified many recurrent HPV-host fusions in CESC, including known HPV integrations at *RAD51B* and *ERBB2* and a novel HPV integration at *PTPN13*.

We compared HBV virus abundance in tumor and paired adjacent normal samples of a large LIHC sample set, failing to find virus enrichment in the tumor, in contrast to the findings for HPV16 in HNSC and CESC, which may represent two different mechanisms of virus-associated tumorigenesis: First, there is a direct carcinogenesis such as what is observed for HPV, in which the virally infected cells directly undergo malignant transformation. The second is the “hit-and-run” scenario where the HBV or HCV-infection triggers inflammation that in turn leads to malignant transformation of neighboring cells without the presence of viral infection. In addition, we found the *X* gene is highly expressed in both tumor and adjacent normal samples. The integration sites in tumor and adjacent normal samples are entirely different. For instance, recurrent HBV integrations in the *KMT2B* (*MLL4*) were observed in tumors, but none in adjacent normal samples. The current study shows that the HBV integration site is a key factor for driving tumorigenesis. The HBV genes may be expressed to sustain infection, but not required for initiation and/or progression of cancer. Lower tumor expression may also be related to evading host immune system response.

HHV4 has been previously shown to be associated with different cancer types, including Hodgkin’s lymphoma, Burkitt’s lymphoma, nasopharyngeal carcinoma^59^ and gastric adenocarcinoma^35^. We observed a significant enrichment of HHVs in the gastrointestinal system, including the known HHV4 carcinogen and more recently identified HHV5. The tumor-adjacent normal pairs provide evidence of enrichment of both HHV4 and HHV5 in tumors. Also, according to the analysis of viral gene expression, we found that viral oncogenes such as *EBER-1* in HHV4 and *RNA2.7* in HHV5 are highly expressed across different tumor samples, supporting likely classifications as virus oncogenes. In addition, the analyses of virus variants in tumor samples reveal the association of virus variants and ethnicity groups for HBV in LIHC and HHV4 in STAD. Finally, we want to note that the current study focuses on known viruses and their current presence on the tumor samples. There are potential novel viruses awaiting discovery, which may also play important roles on the initialization and progression of tumors.

## Materials and Methods

### Twenty-three cancer types included in this study

We collected 7,372 TCGA RNA-Seq data sets from CGHub (https://cghub.ucsc.edu) across 23 cancer types including esophageal cancer (ESCA), stomach, colon, and rectal adenocarcinomas (STAD, COAD, and READ), liver hepatocellular carcinoma (LIHC), pancreatic adenocarcinoma (PAAD), kidney chromophobe (KICH), kidney renal clear cell carcinoma (KIRC), kidney renal papillary cell carcinoma (KIRP), bladder urothelial carcinoma (BLCA), prostate adenocarcinoma (PRAD), uterine carcinosarcoma (UCS), cervical squamous cell carcinoma and endocervical adenocarcinoma (CESC), head/neck squamous cell carcinoma (HNSC), lung squamous cell carcinoma (LUSC), lung adenocarcinoma (LUAD), thyroid carcinoma (THCA), diffuse large B-cell lymphoma (DLBC), acute myeloid leukemia (LAML), sarcoma (SARC), skin cutaneous melanoma (SKCM), glioblastoma multiforme (GBM) and brain lower grade glioma (LGG).

### VirusScan pipeline

For the purpose of constructing a complete virus database, we extracted viral sequences from NCBI NT database (Version: 01/24/2014) and used CD-HIT^60^ to cluster these sequences using a 98% identify cut-off. The clustered sequences form the virus NT database were used in the VirusScan pipeline.

The outline of the VirusScan pipeline is illustrated in Fig. S1 and includes the following steps. First, unmapped reads and reads poorly mapped to human genome from the input bam files were extracted. Next “BWA –aln” (version: 0.6.1-r104)^61^ was used to align the extracted reads obtained in step 1) to the Virus NT database, with potential viral hits proceeding to the following analysis. Finally, repetitive sequences were marked and sequence quality control was performed. Many eukaryotic genomes contain large batches of highly repetitive DNA sequences, which causes problems in BLAST-based similarity searches and results in high rates of false-positive alignments. RepeatMasker (http://www.repeatmasker.org) was used to mask interspersed repeats and low complexity DNA sequences. A sequence failed the quality control criteria if it does not contain a stretch of at least 40 consecutive non-“N” nucleotides (i.e., “Filtered sequence”) or if greater than 40% of the total length of the sequence is masked (i.e., “low complexity sequence”). These sequences were removed from further analysis.

1. Further filter human sequences by using MegaBlast against human genome and transcript database.
2. Run MegaBlast against NCBI NT database for the remaining reads after filtering human sequences.
3. Use NCBI taxonomy database to classify viruses and BLAST results to get the specific viral species.

The VirusScan pipeline is written in Perl and incorporates many standard software packages, such as BWA, RepeatMasker, and the NCBI BLAST suite. The pipeline is fully automated and can run multiple jobs in parallel on a high performance compute cluster, developing from VirusHunter pipeline. In contrast to VirusHunter^62^, which is designed primarily for the discovery of novel viruses, VirusScan focuses the fast identification of known viruses. Benchmark testing shows ~500 RNA-Seq bams processed in one day using 200 CPUs (Intel(R) Xeon(R) X5660 at 2.80 GHz). We note that other bioinformatics tools exist for the detection of viruses or the integration sites^29^,^63^,^64^,^65^,^66^,^67^,^68^.

### Threshold for virus-positive samples

With a large collection of cancer samples having high prevalences of HPV, such as HNSC, CESC and BLCA, etc., we have the power to examine the distribution of HPV abundance across all tumor samples. Figure S2 shows the histogram of the number of samples vs HPV’s RPHM. We found a bimodal distribution for HPV’s abundance. There are two clusters of samples, one is RPHM ≤ 10^2^ and the other is RPHM ≥ 10^3^. The observed two clusters may represent two sets of different samples, i.e., one includes samples with low-level HPV contamination or in latent stage of HPV infection and the other consists of samples with actively transcribed HPV. The bimodal distribution suggests that RPHM ≥ 10^2^ is a reasonable cut-off for defining virus-positive samples.

### Viral gene expressions

For estimating viral gene expressions and virus integrations, we created a custom reference sequence consisting of the GRCh37 human reference, together with virus types identified during RNA-Seq processing, and realigned all RNA-Seq data to this reference using BWA.

Virus gene expression was assessed. Virus gene annotations were downloaded from NCBI. Sequences were aligned against the viral references using BWA and read counts for the targeted viral genes were obtained. Read counts were scaled to report the number of viral reads per 100 million total sequence reads.

### Integration sites

We use BWA and Pindel to identify the putative virus integration sites. The detailed steps are as following:

1. Extracted pair-end reads from imported bam files, in which reads are aligned to human sequence.
2. Use “BWA sampe” to align the extracted pair-end reads to human plus virus reference. The aligned files were evaluated for presence of human-virus discordant reads, where one read of a read pair maps to human, the other to virus. Such discordant read pair clusters correspond to break points and likely viral integration sites.
3. Run Pindel^48^ on the samples with ≥10 human-virus discordant reads to identify putative breakpoints based on read pair analysis.
4. Look *RP file from Pindel output directory for putative integration sites; see Supplementary Tables 3–5 for HPV integration sites on CESC and HNSC and HBV integration sites on LIHC samples.
5. Intersect with Ensembl 37.75 gene annotation file and select host genes with ≥10 supporting discordant human-virus pairs for recurrent virus-host integration analysis at gene level (see Fig. 4A).

### Exon-level host gene expression

We generated RPKM value for the exons of different genes based on the TCGA RNA-Seq bams. The detailed steps are as follows. First, bed files were generated for the exon boundary based on Ensembl 37.75 database, followed by the use of the “bedtools -multicov –bam input.bam –bed input.bed” command to count raw reads for all exons. In the final step RPKM based on RPKM = (10^9^ * R)/(N * L) was calculated where R is the number of raw read mapped to the exon and N is the total mapped reads and L is the length of the exon.

### Phylogenetic Analysis

We used the contml tool from the PHYLIP toolkit (http://evolution.genetics.washington.edu/phylip.html) to construct phylogenetic trees based on variant allele fraction for HBV and HHV4. We eliminated duplicate samples, as PHYLIP does not allow such processing. Each analysis used random input of the order of the samples. Output in NEWICK format was used in iTOL (itol.embl.de) to visualize results.

## Additional Information

**How to cite this article**: Cao, S. *et al*. Divergent viral presentation among human tumors and adjacent normal tissues. *Sci. Rep.* **6**, 28294; doi: 10.1038/srep28294 (2016).

## Supplementary Material

Supplementary Information

Supplementary Dataset 1

Supplementary Dataset 2

Supplementary Dataset 3

Supplementary Dataset 4
