# Supplementary material for: Divergent viral presentation among human tumors and adjacent normal tissues
Source: Sci Rep. 2016 Jun 24;6:28294. doi: 10.1038/srep28294 (PMC4919655; doi:10.1038/srep28294)
Supplement: Supplementary Information [file srep28294-s1.pdf]

Supplementary materials for **"Divergent viral presentation among human tumors and adjacent normal tissues"**

Song Cao[1], Michael C. Wendl[1,3,5], Matthew A. Wyczalkowski[1], Kristine Wylie[1,6], Kai Ye[1,3], Reyka Jayasinghe[1,2], Mingchao Xie[1,2], Song Wu[1], Beifang Niu[1], Robert Grubb III[7], Kimberly J. Johnson[8], Hiram Gay[4], Ken Chen[9], Janet S. Rader[10], John F. Dipersio[2,4], Feng Chen[2,4], and Li Ding[1,2,3,4#]

McDonnell Genome Institute[1], Department of Medicine[2], Department of Genetics[3], Siteman Cancer Center[4], Department of Mathematics[5], Department of Pediatrics[6], Department of Surgery[7], Washington University, St. Louis, Missouri 63108, USA

[8]Brown School Master of Public Health Program, Washington University in St. Louis, St. Louis, MO 63130, USA

[9]The University of Texas MD Anderson Cancer Center, Department of Bioinformatics and Computational Biology, Houston, Texas 77030, USA

[10]Department of Obstetrics and Gynecology, Medical College of Wisconsin, Milwaukee, WI 53226, USA

# Corresponding Author:
Li Ding, Ph.D
McDonnell Genome Institute
Division of Oncology, Department of Medicine
Washington University School of Medicine
St. Louis, MO 63108
Email: lding@genome.wustl.edu

Virus discovery of CESC and other cancer types

The vast majority of CESC samples are HPV-positive (~92%) having RPHM≥100, with 16 different subtypes observed. HPV16 represents 57.6% of HPV positive cases, followed by HPV18 and HPV45, with 12.7% and 7.1%, respectively. HPV31, HPV33, HPV35, HPV39, and HPV52 are 2.4%, 2.4%, 1.6%, 1.6% and 2.4%, respectively (Fig. 1A). High HPV prevalence in CESC is consistent with a meta-study using a PCR-based HPV assay[1] and RNA-Seq data[2,3]. However, using a complete nucleotide reference database, we were able to further detect several rare HPV subtypes, such as HPV26 and HPV30, which were not reported previously[1,2,4,5] (Fig. 1A).

Our analysis shows a small fraction of virus-positive samples in other cancer types such as pancreatic adenocarcinoma (PAAD), kidney renal clear cell carcinoma (KIRC), bladder urothelial carcinoma (BLCA), uterine carcinosarcoma (UCS), lung squamous cell carcinoma (LUSC), sarcoma (SARC), skin cutaneous melanoma (SKCM) and brain lower grade glioma (LGG) (Fig. 1A). For instance, in BLCA, we found one sample with BK virus, two samples with HPV16, five with HPV11, HPV45, HPV52, HPV56 or HPV6 and four with HHV5, largely consistent with a previous TCGA report[6]. Levels for BK, HPV16, HPV52, and HPV6 are very high (RPHM>10^4) (Fig. S3C). We found three LGG samples with HPV16 and one sample with HPV58. The RPHMs of HPV16 are between 10^2 and 10^3 (Fig. S3D and S9) and E6 and E7 are expressed at higher levels than other viral genes in LGG. In addition, we identified one LUSC and one SKCM with a very high abundance of HHV4 (Fig. 1A and Fig. S3B). The findings of HPV16 on LGG and HHV4 on LUSC and SKCM indicate that HPV16 and HHV4 may play important role in other cancer types.

Reference:

1. Tang KW, Alaei-Mahabadi B, Samuelsson T, Lindh M, & Larsson E (2013) The landscape of viral expression and host gene fusion and adaptation in human cancer. *Nat Commun* 4:2513.
2. Khoury JD*, et al.* (2013) Landscape of DNA virus associations across human malignant cancers: analysis of 3,775 cases using RNA-Seq. *J Virol* 87(16):8916-8926.
3. Clifford G, Franceschi S, Diaz M, Munoz N, & Villa LL (2006) HPV type-distribution in women with and without cervical neoplastic diseases. *Vaccine* 24:26-34.
4. Ojesina AI*, et al.* (2014) Landscape of genomic alterations in cervical carcinomas. *Nature* 506(7488):371-375.
5. Geraets D*, et al.* (2012) Detection of rare and possibly carcinogenic human papillomavirus genotypes as single infections in invasive cervical cancer. *J Pathol* 228(4):534-543.
6. Anonymous (2014) Comprehensive molecular characterization of urothelial bladder carcinoma. *Nature* 507(7492):315-322.

Supplementary figures

Supplementary Figure 1 (Page 6): The schematics of VirusScan pipeline.

Supplementary Figure 2 (Page 7): The histogram of HPV's RPHM among all tumor samples.

Supplementary Figure 3 (Pages 8-11): The abundance (RPHM) of viruses detected in (a) gastrointestinal cancers, (b) gynecologic and head and neck cancers , (c) bladder and kidney cancers and (d) other cancer types. We use different colors to illustrate the virus abundance in log10 scale. In the figure, we only show viruses with RPHM>=100.

Supplementary Figure 4 (Page 12): RNA-Seq read counts for HHVs across gastrointestinal cancers.

Supplementary Figure 5 (Page 13): Read depths for HHV4 in samples with RPHM>=100 across whole virus genome.

Supplementary Figure 6 (Page 14): Read depths for HHV5 in samples with RPHM>=100 across the whole virus genome.

Supplementary Figure 7 (Pages 15-16): Read depths for (a) HHV1 and (b) HHV6 in samples with RPHM>=100 across the whole virus genome.

Supplementary Figure 8 (Pages 17-18): Unsupervised clustering of samples based on the gene expression of (a) HPV16 and (b) HPV18.

Supplementary Figure 9 (Page 19): Read depths for HPV16 in the entire virus genome across three LGG samples.

Supplementary Figure 10 (Page 20): Comparison of *MYC* expression for samples with HPV integrations on *CASC8* and *PVT1* and samples without these integration sites.

Supplementary Figure 11 (Pages 21-22): The RPKM's difference between the case and the mean value of controls for different exons in the longest transcripts of (a) genes CTSE, GRHL2, CD274 and RAD51B and (b) genes RNF166, ABR, PGAP3 and IKZF3 with recurrent virus integrations. We use the circle's size to quantify the –log(P), which P is the p-value of the case significant from the controls. Different samples are marked by different colors.

Supplementary Figure 12 (Page 23): (a) The number of virus variants and (b) the mutation rate for these viruses across different samples.

Supplementary Figure 13 (Page 24): The frequency of HPV16 variants across HNSC (Gray) and CESC (Blue) samples.

Supplementary Figure 14 (Page 25): Unsupervised clustering of samples based on HPV16 variants in (a) HNSC and (b) CESC. All the selected variant sites have a coverage higher than 10 across all samples.

Supplementary Tables:

Supplementary Table 1 (Pages 26-27): The abbreviations of cancer types, viruses and human and viral gene names.

Supplementary Table 2 (Pages 28-34): The location of common integration sites found in CESC, HNSC and LIHC.

Fig. S1

VirusScan Pipeline

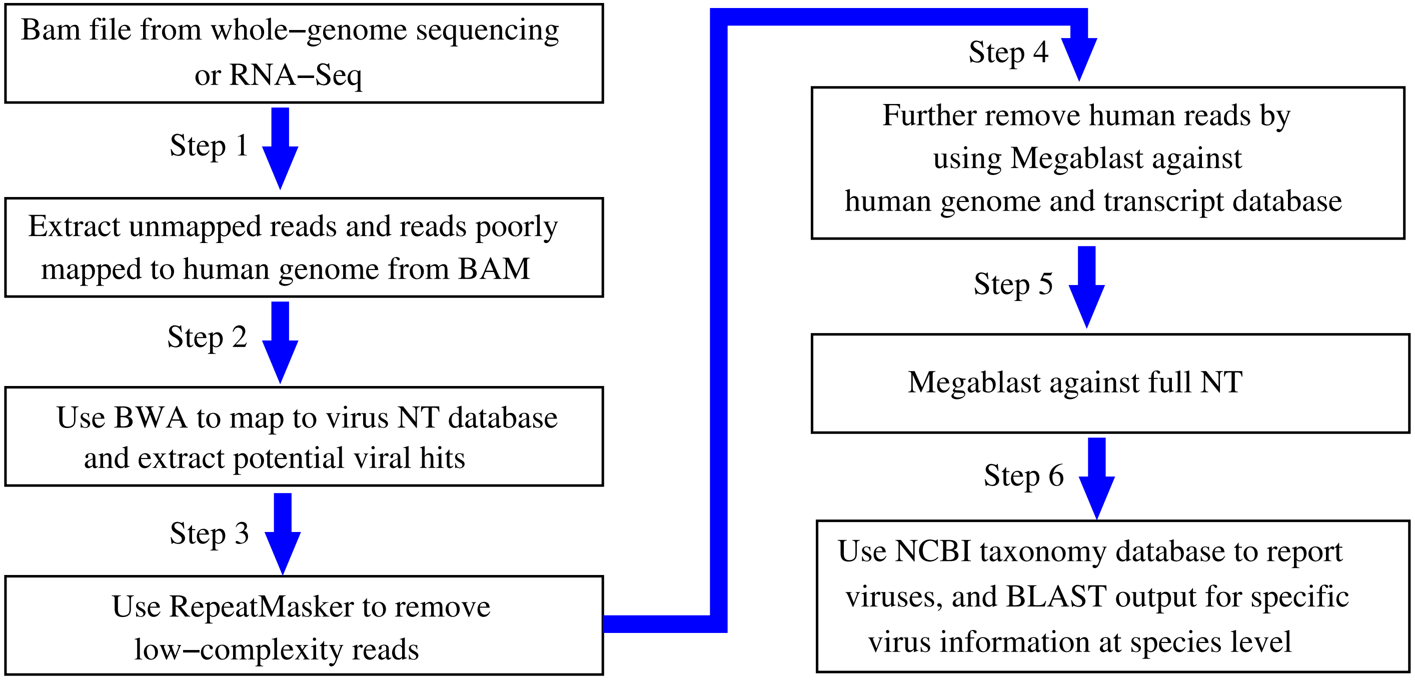

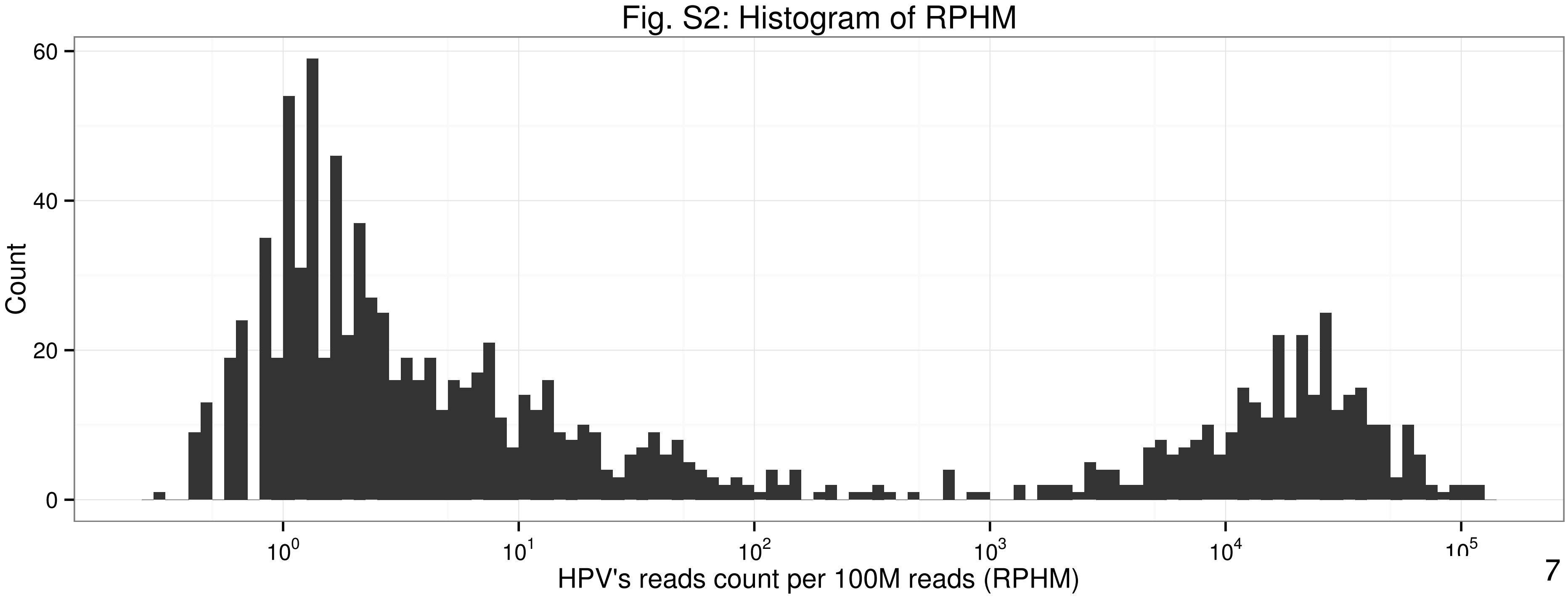
Fig. S2: Histogram of RPHM
Count
60
40
20
0
10^0
10^1
10^2
10^3
10^4
10^5
HPV's reads count per 100M reads (RPHM)

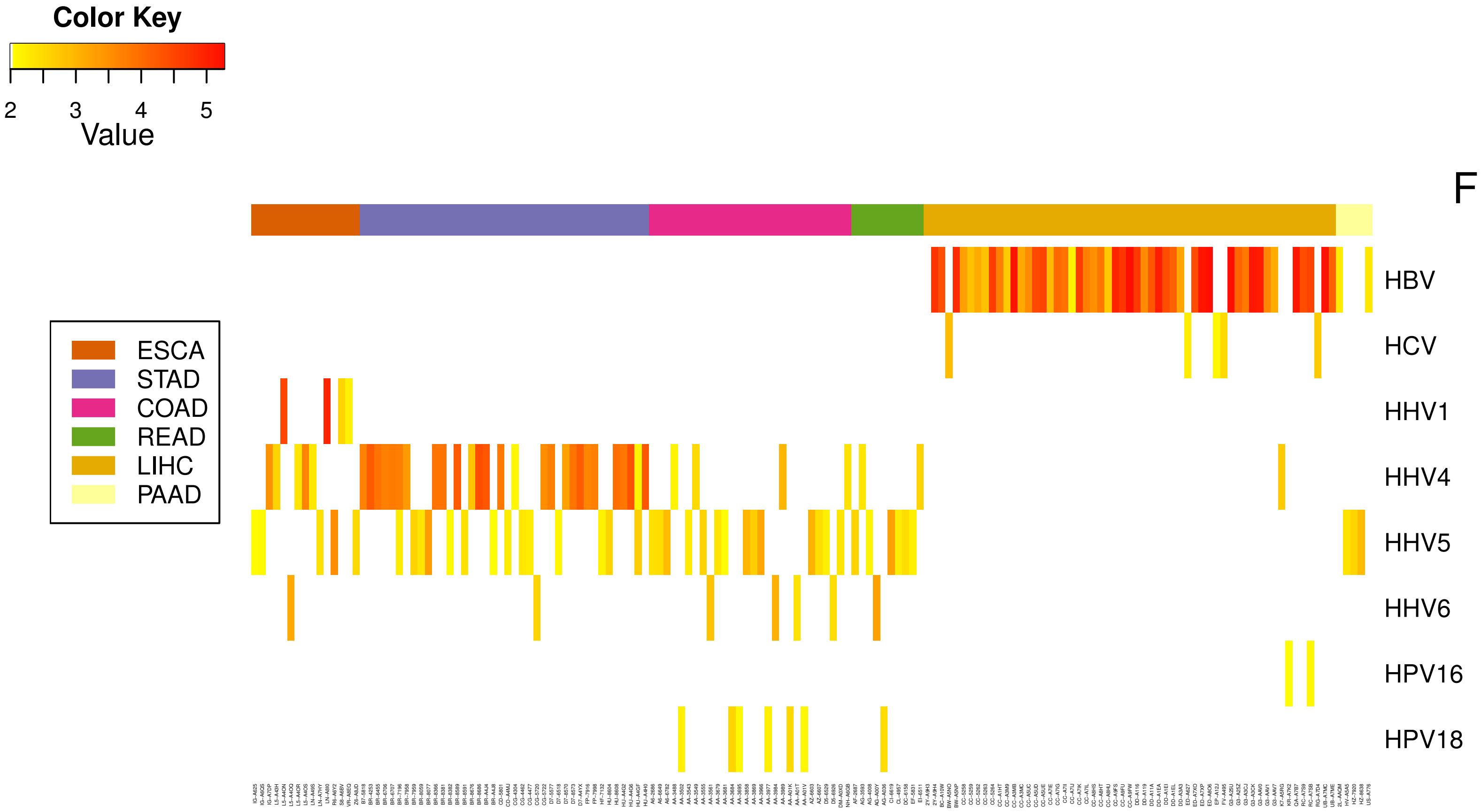

Color Key
2
3
4
5
Value
ESCA
STAD
COAD
READ
LIHC
PAAD
HBV
HCV
HHV1
HHV4
HHV5
HHV6
HPV16
HPV18

Fig. S3a

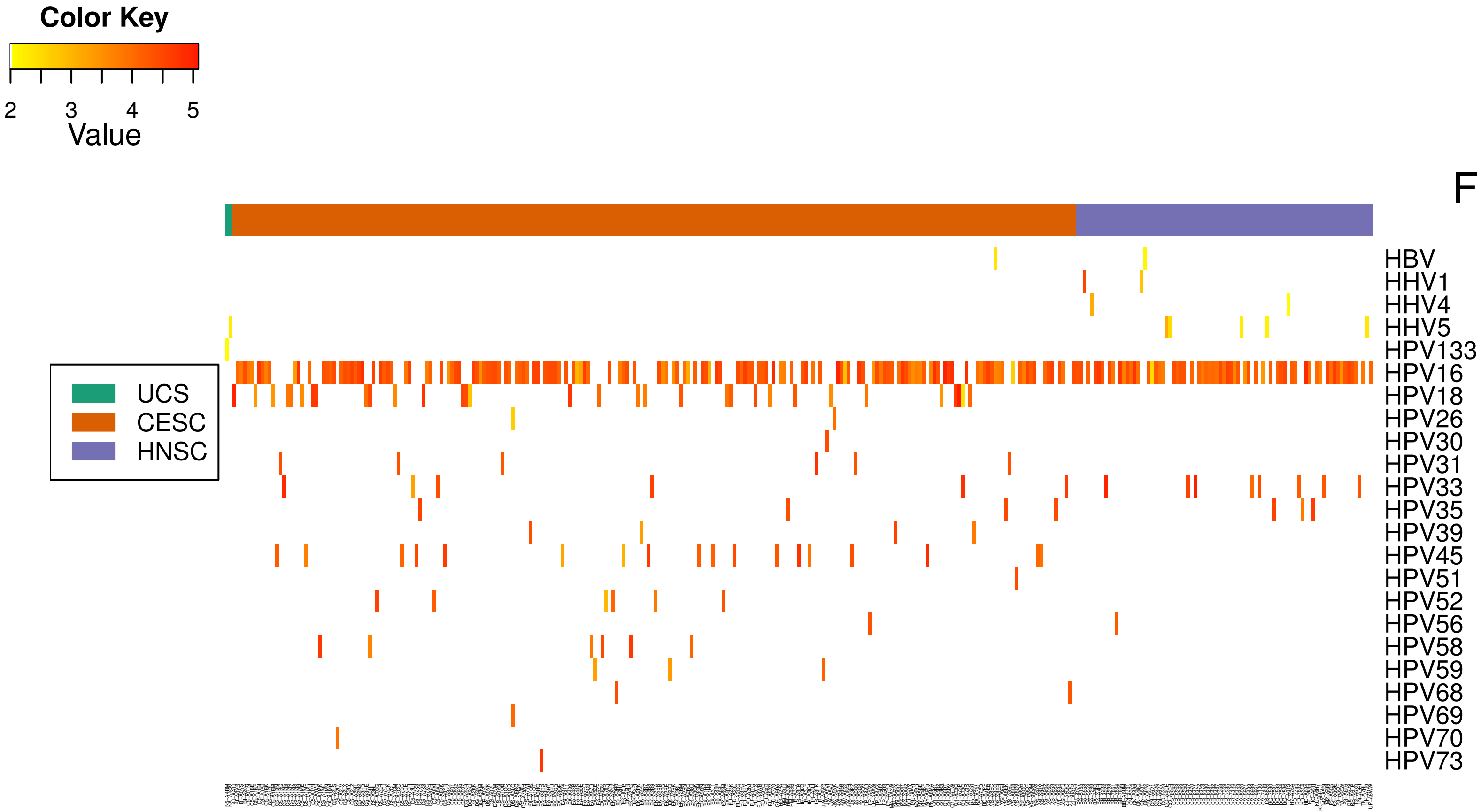
Color Key
2
3
4
5
Value
UCS
CESC
HNSC
HBV
HHV1
HHV4
HHV5
HPV133
HPV16
HPV18
HPV26
HPV30
HPV31
HPV33
HPV35
HPV39
HPV45
HPV51
HPV52
HPV56
HPV58
HPV59
HPV68
HPV69
HPV70
HPV73

Fig. S3b

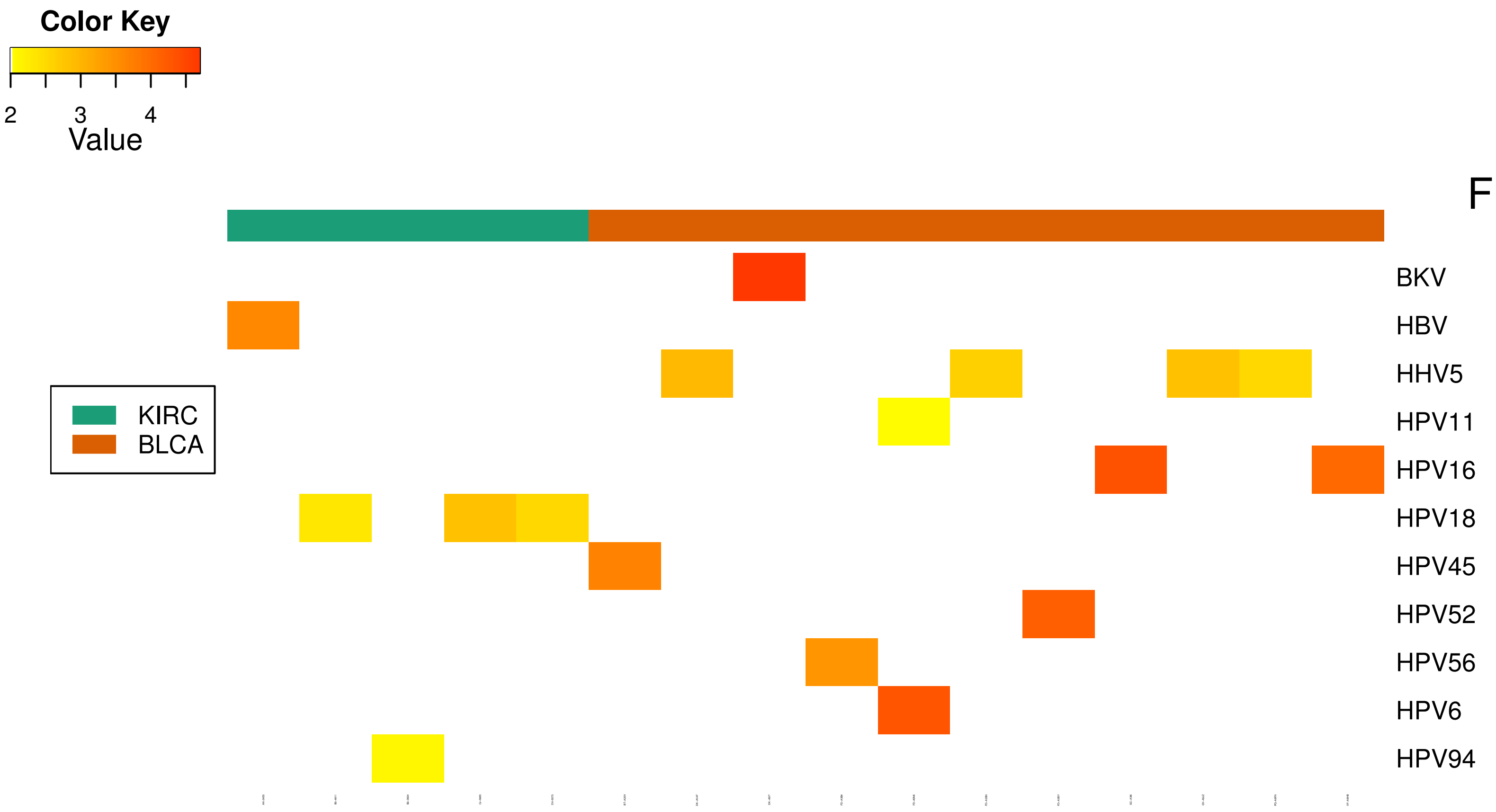
Color Key
2
3
4
Value
KIRC
BLCA
BKV
HBV
HHV5
HPV11
HPV16
HPV18
HPV45
HPV52
HPV56
HPV6
HPV94
Fig. S3c

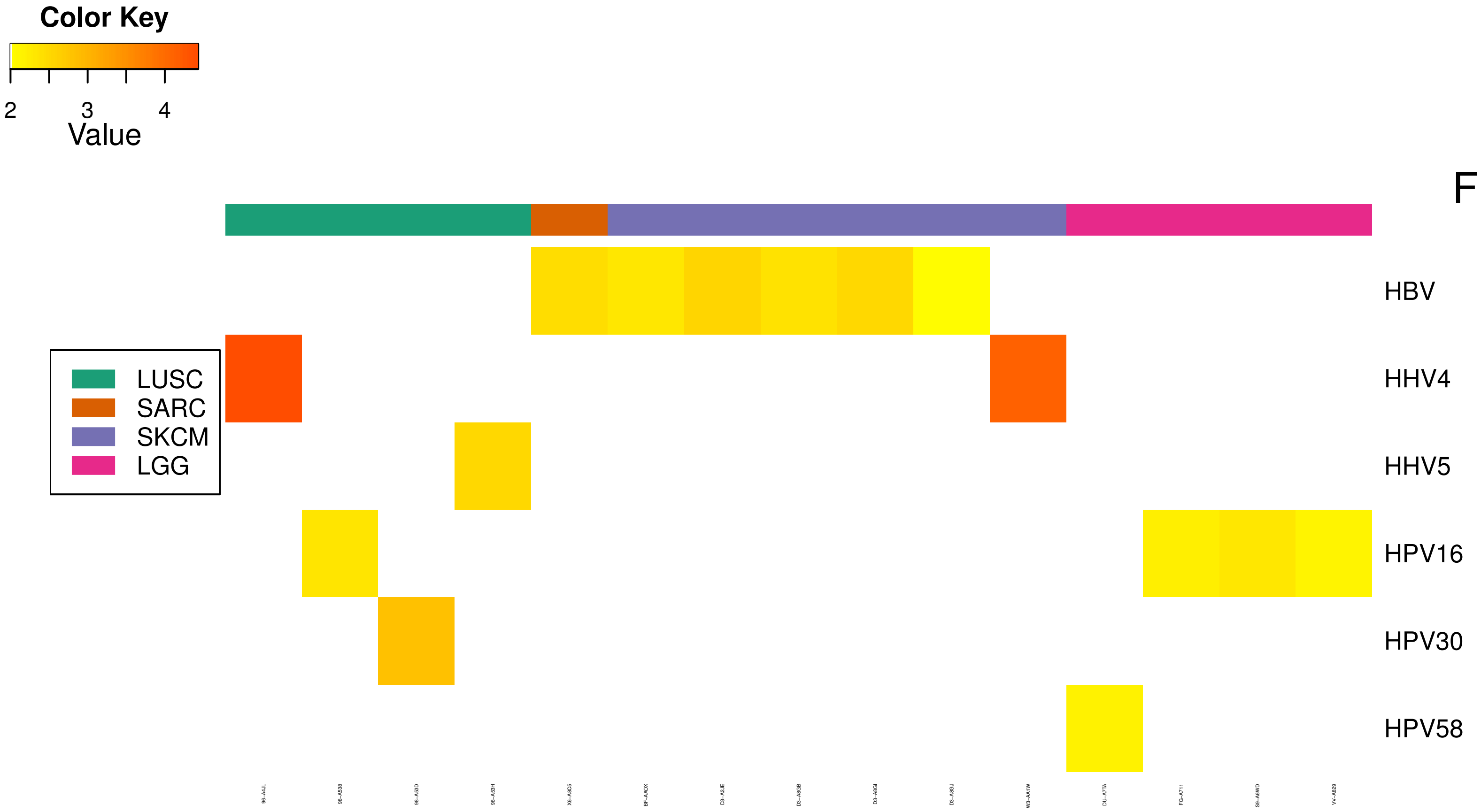

Color Key
2
3
4
Value
LUSC
SARC
SKCM
LGG
HBV
HHV4
HHV5
HPV16
HPV30
HPV58
96–A4JL
98–A538
98–A53D
98–A53H
X6–A8C5
BF–AAOX
D3–A2JE
D3–A8GB
D3–A8GI
D3–A8GJ
W3–AA1W
DU–A7TA
FG–A711
S9–A6WD
VV–A829

Fig. S3d

Fig. S4: Histogram of HHVs RNA–Seq Read Counts

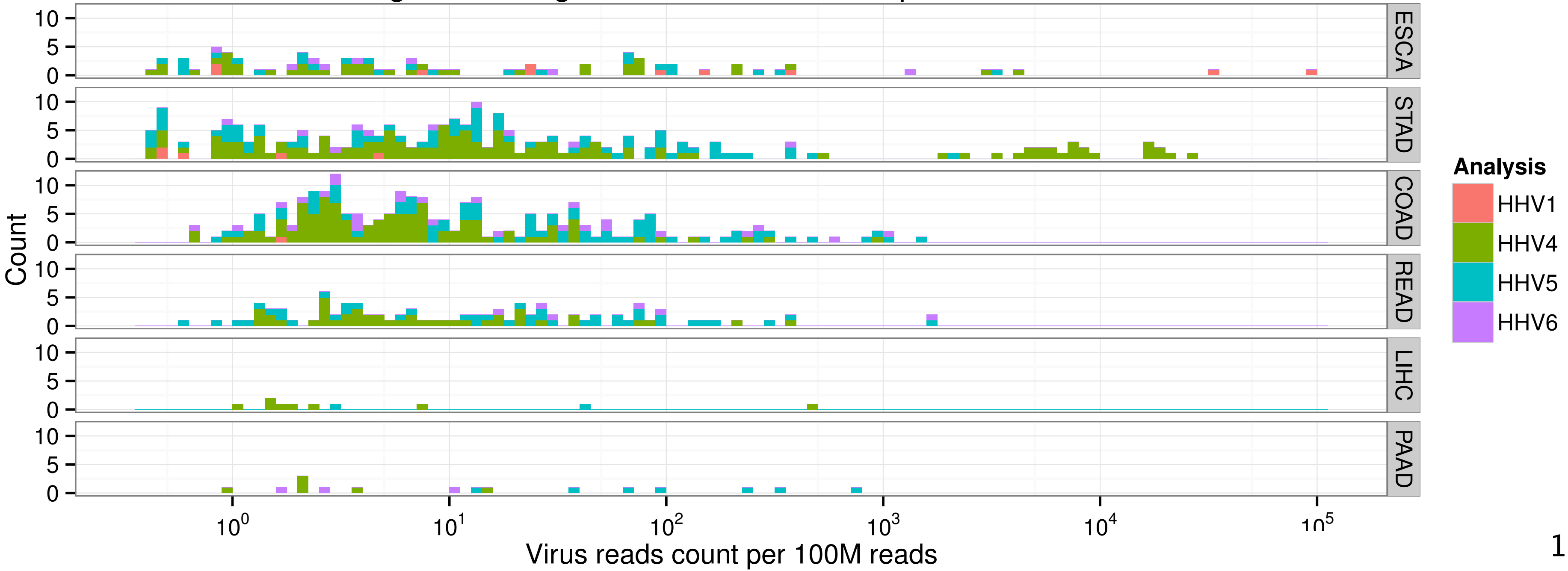

Fig. S5

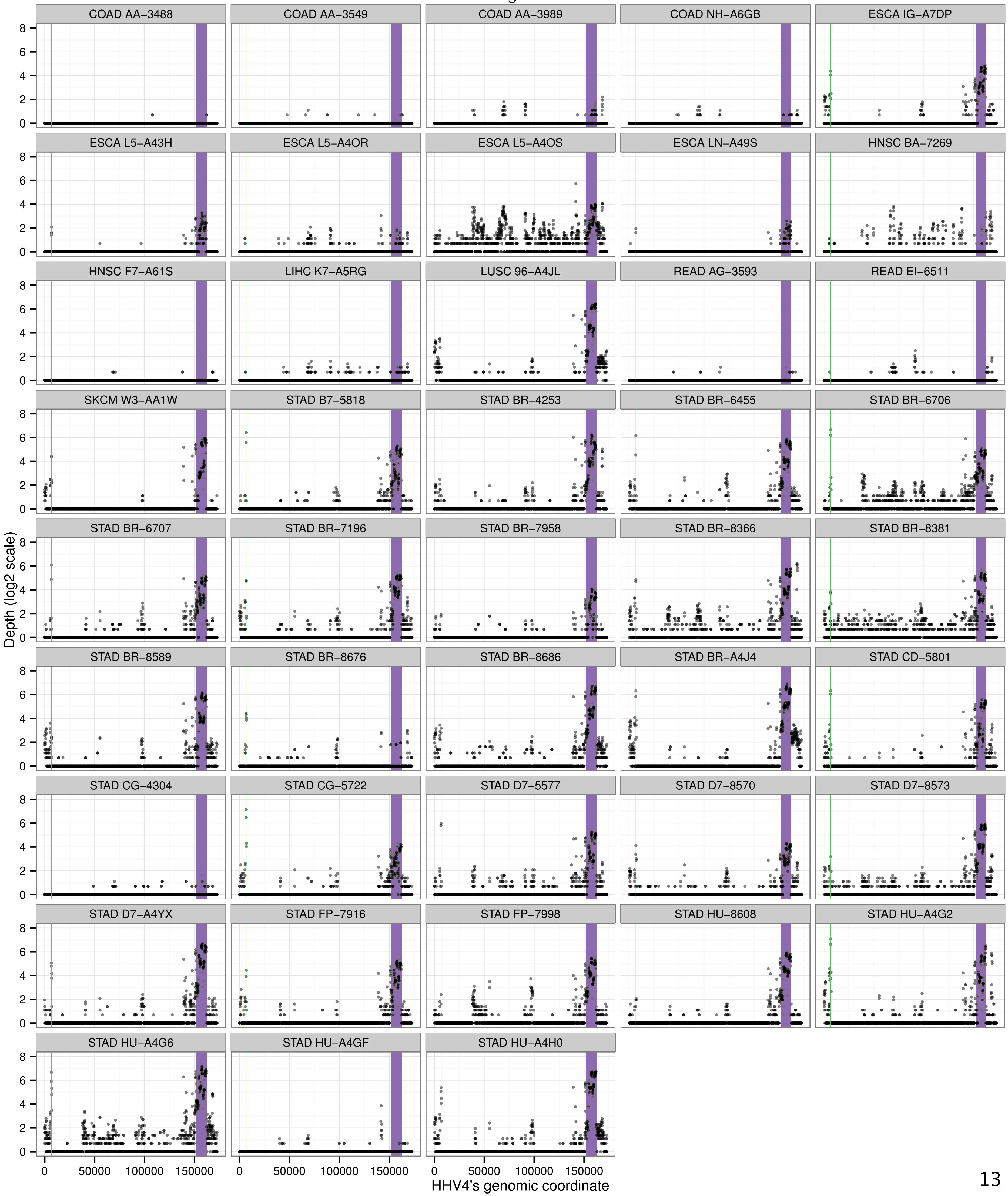
COAD AA−3488
COAD AA−3549
COAD AA−3989
COAD NH−A6GB
ESCA IG−A7DP
ESCA L5−A43H
ESCA L5−A4OR
ESCA L5−A4OS
ESCA LN−A49S
HNSC BA−7269
HNSC F7−A61S
LIHC K7−A5RG
LUSC 96−A4JL
READ AG−3593
READ EI−6511
SKCM W3−AA1W
STAD B7−5818
STAD BR−4253
STAD BR−6455
STAD BR−6706
STAD BR−6707
STAD BR−7196
STAD BR−7958
STAD BR−8366
STAD BR−8381
STAD BR−8589
STAD BR−8676
STAD BR−8686
STAD BR−A4J4
STAD CD−5801
STAD CG−4304
STAD CG−5722
STAD D7−5577
STAD D7−8570
STAD D7−8573
STAD D7−A4YX
STAD FP−7916
STAD FP−7998
STAD HU−8608
STAD HU−A4G2
STAD HU−A4G6
STAD HU−A4GF
STAD HU−A4H0
Depth (log2 scale)
0
2
4
6
8
0
50000
100000
150000
HHV4's genomic coordinate

Fig. S6

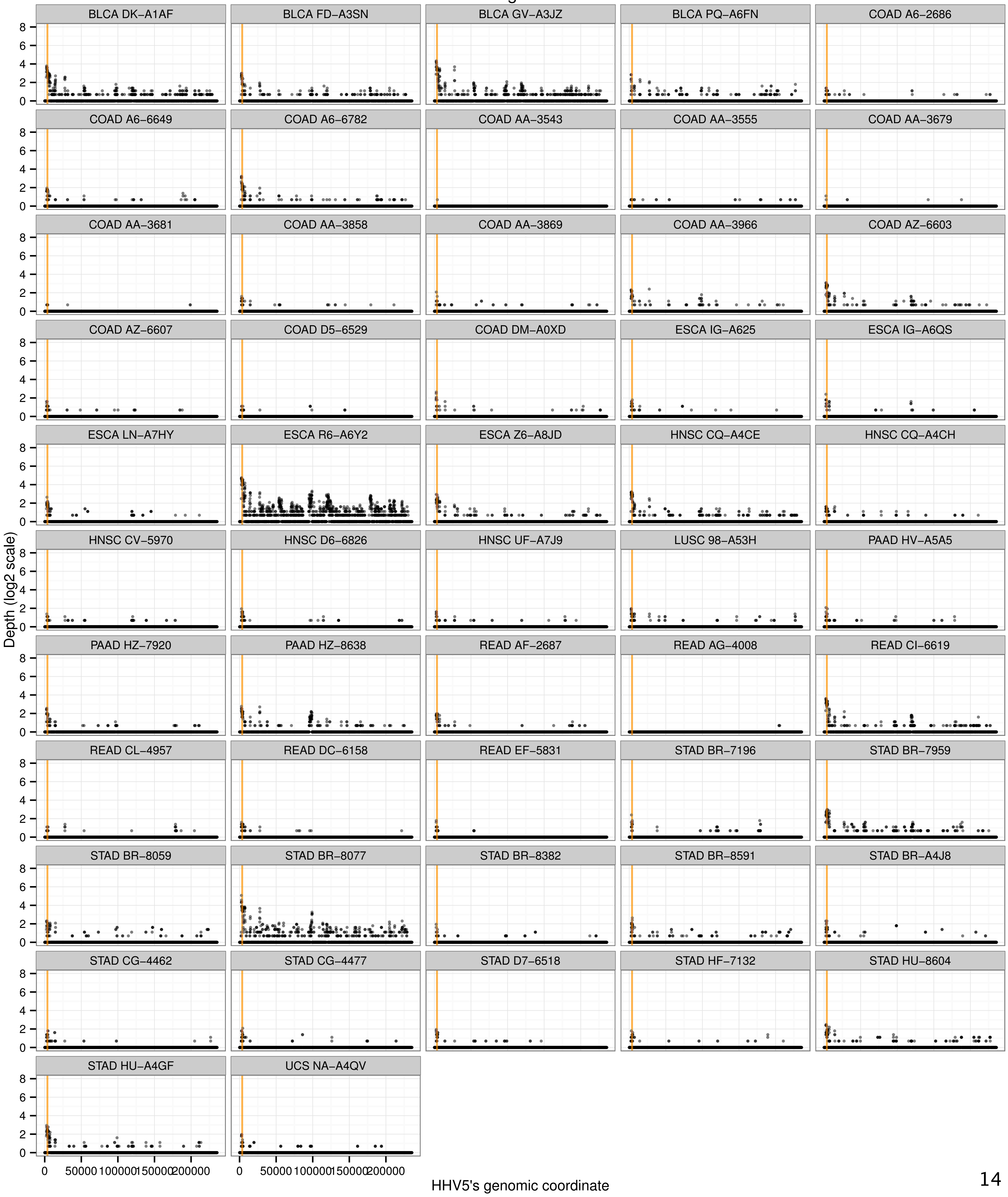

BLCA DK–A1AF
BLCA FD–A3SN
BLCA GV–A3JZ
BLCA PQ–A6FN
COAD A6–2686
COAD A6–6649
COAD A6–6782
COAD AA–3543
COAD AA–3555
COAD AA–3679
COAD AA–3681
COAD AA–3858
COAD AA–3869
COAD AA–3966
COAD AZ–6603
COAD AZ–6607
COAD D5–6529
COAD DM–A0XD
ESCA IG–A625
ESCA IG–A6QS
ESCA LN–A7HY
ESCA R6–A6Y2
ESCA Z6–A8JD
HNSC CQ–A4CE
HNSC CQ–A4CH
HNSC CV–5970
HNSC D6–6826
HNSC UF–A7J9
LUSC 98–A53H
PAAD HV–A5A5
PAAD HZ–7920
PAAD HZ–8638
READ AF–2687
READ AG–4008
READ CI–6619
READ CL–4957
READ DC–6158
READ EF–5831
STAD BR–7196
STAD BR–7959
STAD BR–8059
STAD BR–8077
STAD BR–8382
STAD BR–8591
STAD BR–A4J8
STAD CG–4462
STAD CG–4477
STAD D7–6518
STAD HF–7132
STAD HU–8604
STAD HU–A4GF
UCS NA–A4QV
Depth (log2 scale)
0
2
4
6
8
0
50000
100000
150000
200000
HHV5's genomic coordinate

Fig. S7a

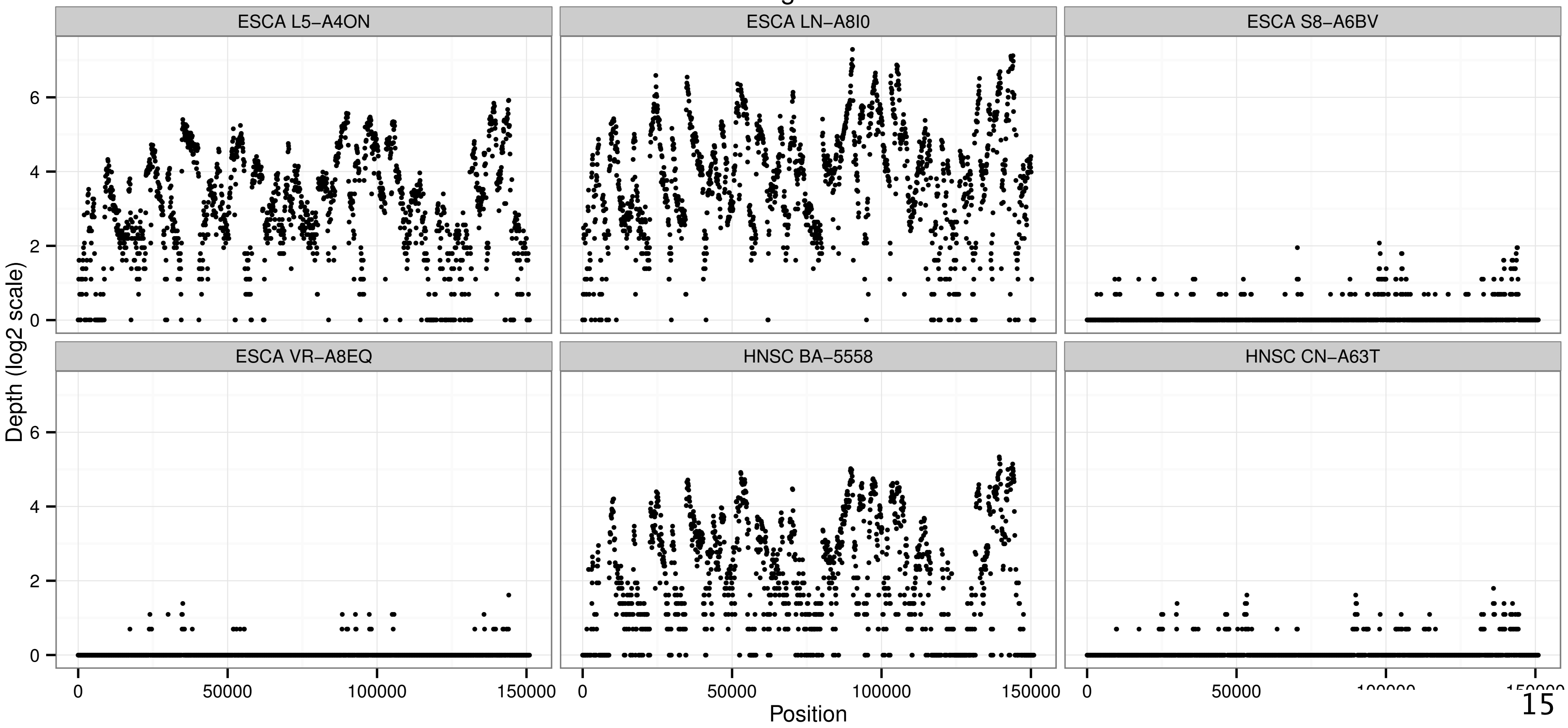
ESCA L5−A4ON
ESCA LN−A8I0
ESCA S8−A6BV
ESCA VR−A8EQ
HNSC BA−5558
HNSC CN−A63T
Depth (log2 scale)
Position
0
2
4
6
0
50000
100000
150000

Fig. S7b

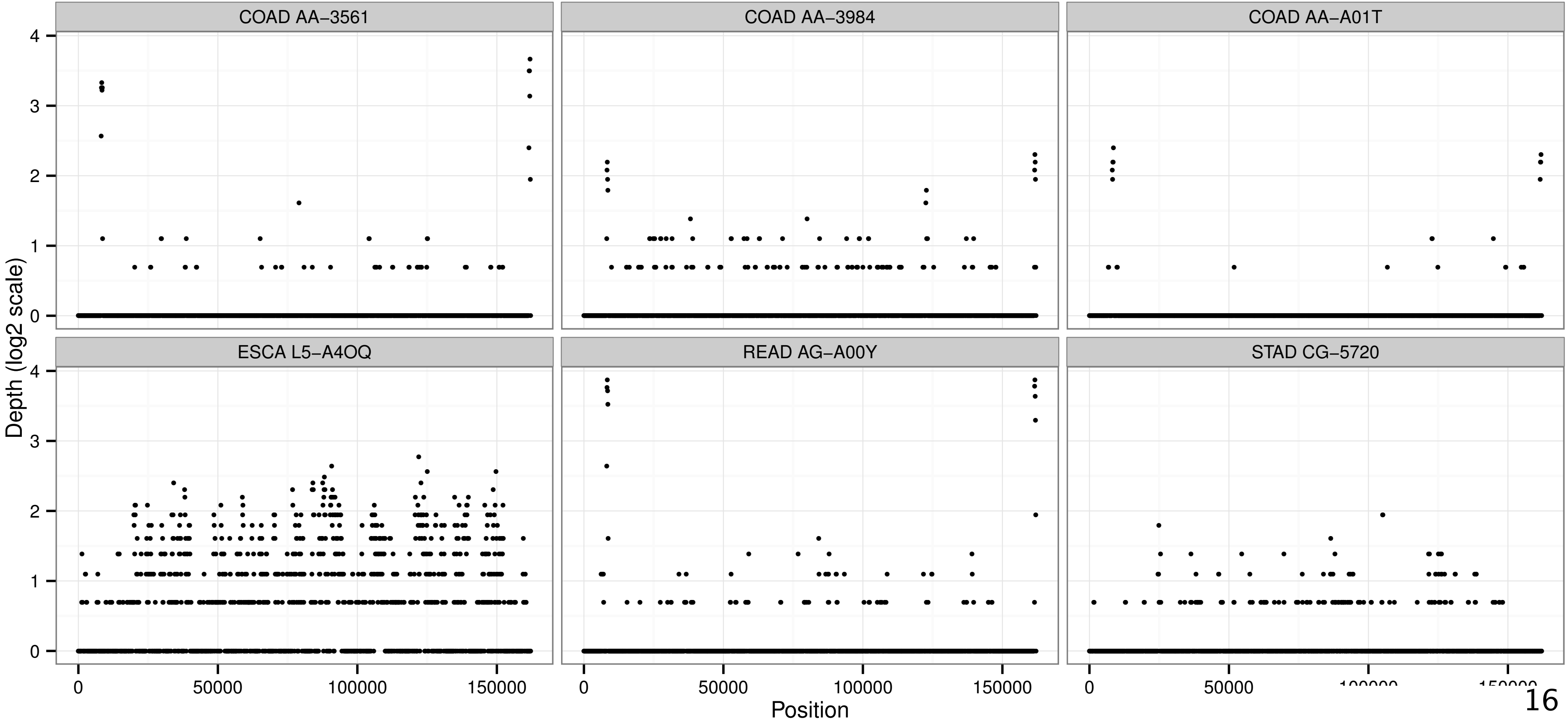

COAD AA−3561
COAD AA−3984
COAD AA−A01T
ESCA L5−A4OQ
READ AG−A00Y
STAD CG−5720
Depth (log2 scale)
0
1
2
3
4
0
50000
100000
150000
Position

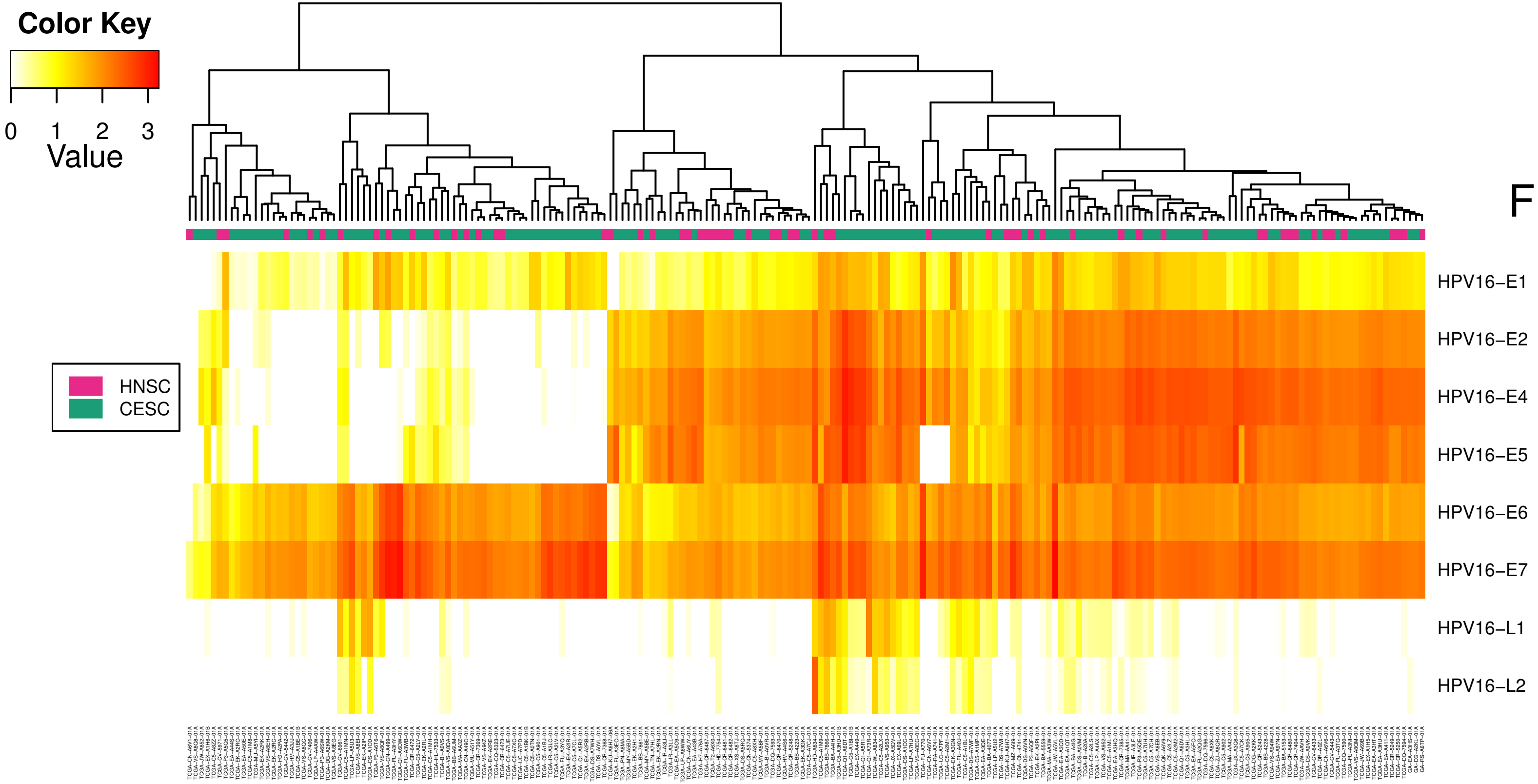
Color Key
0
1
2
3
Value
HNSC
CESC
HPV16–E1
HPV16–E2
HPV16–E4
HPV16–E5
HPV16–E6
HPV16–E7
HPV16–L1
HPV16–L2

Fig. S8a

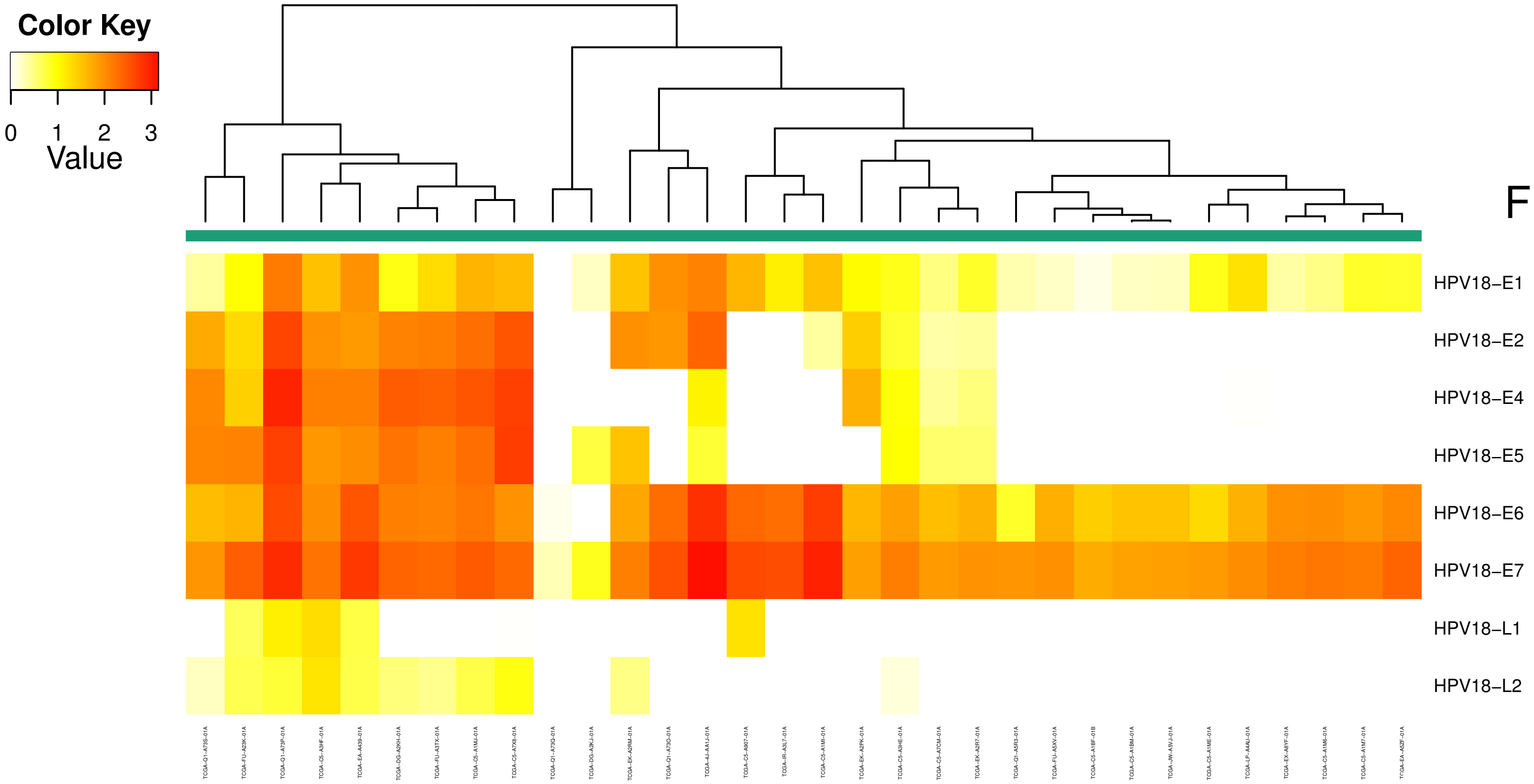
Color Key
0 1 2 3
Value
HPV18–E1
HPV18–E2
HPV18–E4
HPV18–E5
HPV18–E6
HPV18–E7
HPV18–L1
HPV18–L2

Fig. S8b

Fig. S9: LGG Samples with HPV16's RPHM>=100

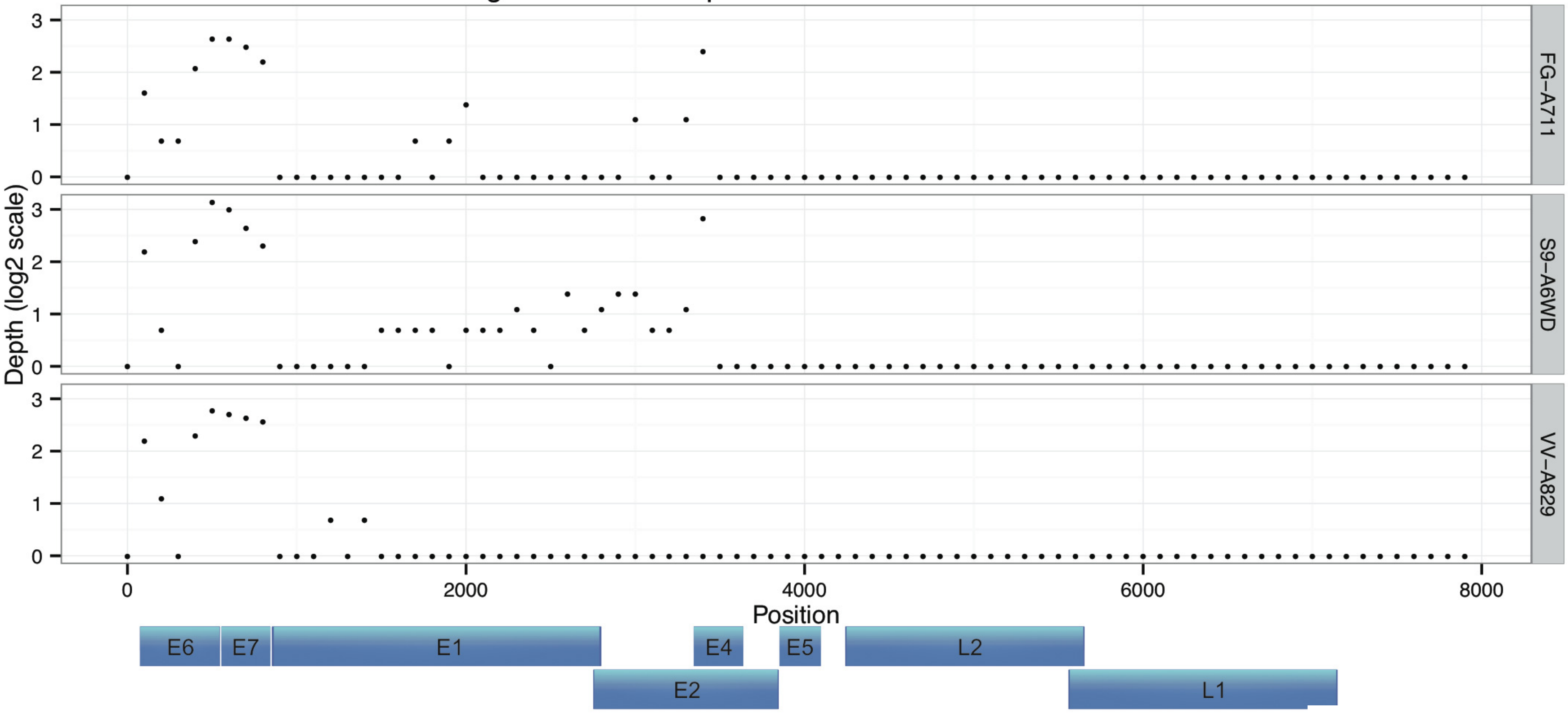

Fig. S10

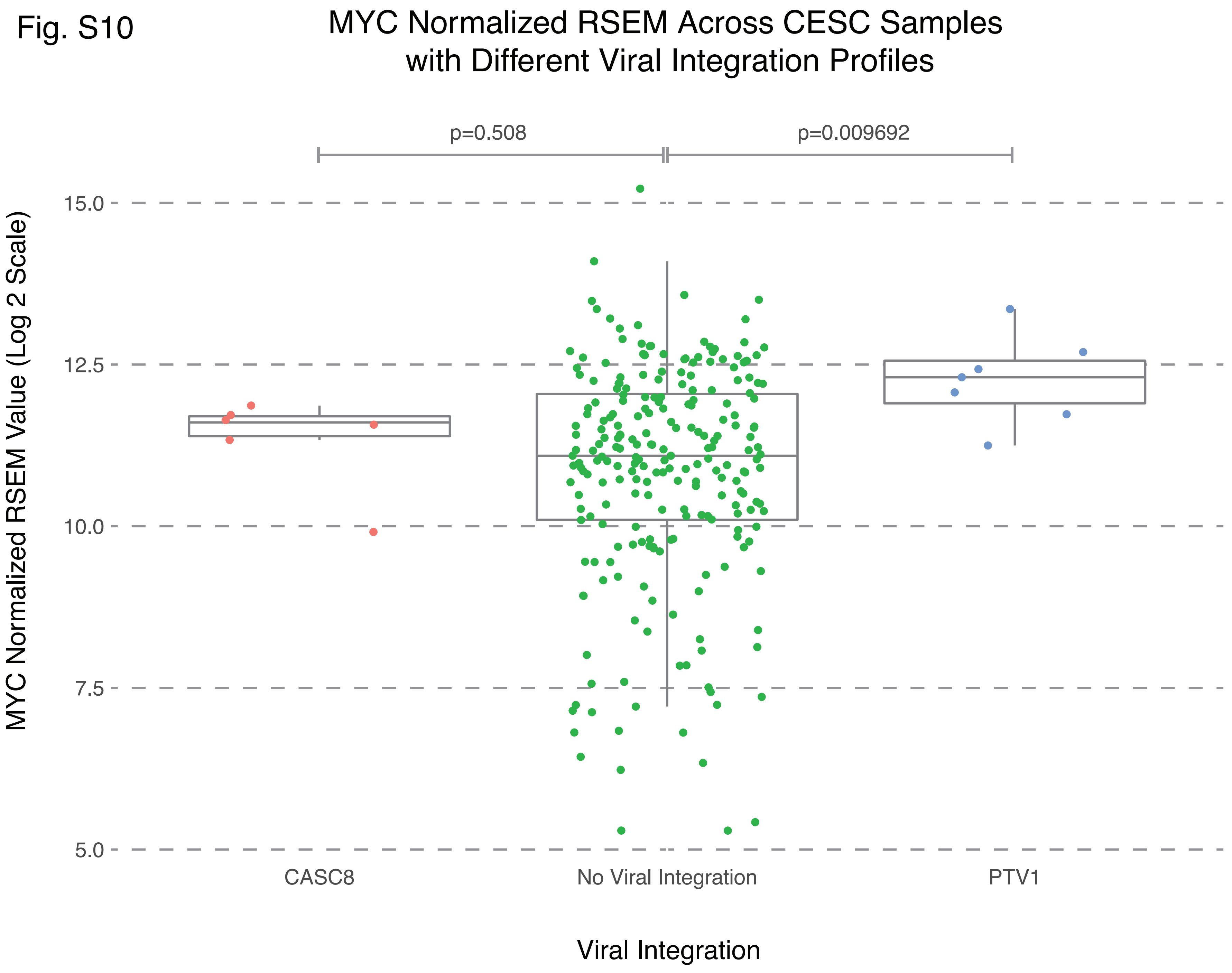
MYC Normalized RSEM Across CESC Samples
with Different Viral Integration Profiles
p=0.508
p=0.009692
MYC Normalized RSEM Value (Log 2 Scale)
15.0
12.5
10.0
7.5
5.0
CASC8
No Viral Integration
PTV1
Viral Integration

Fig. S11a

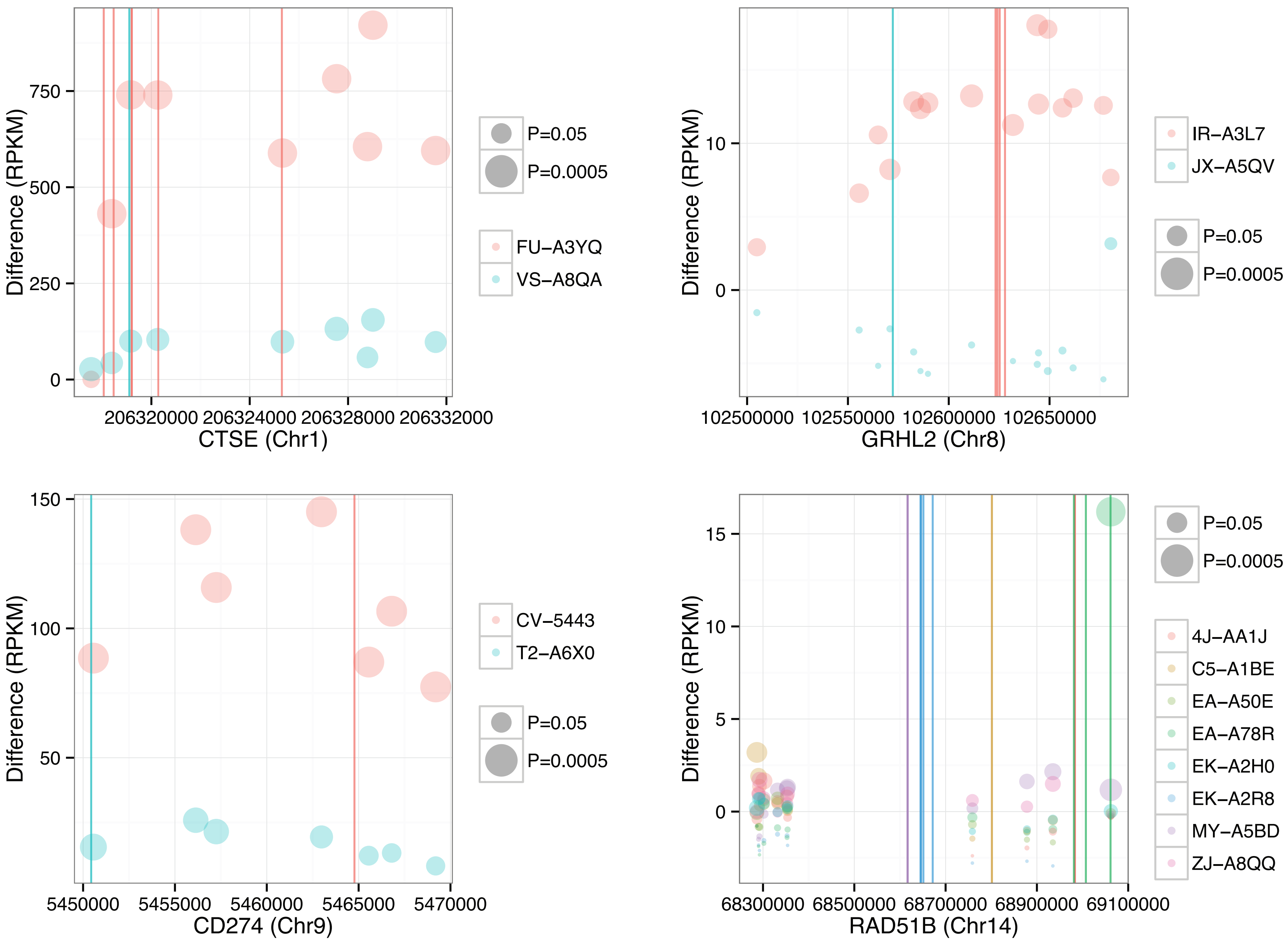

Difference (RPKM)
750
500
250
0
206320000 206324000 206328000 206332000
CTSE (Chr1)
P=0.05
P=0.0005
FU−A3YQ
VS−A8QA
Difference (RPKM)
10
0
102500000 102550000 102600000 102650000
GRHL2 (Chr8)
IR−A3L7
JX−A5QV
P=0.05
P=0.0005
Difference (RPKM)
150
100
50
5450000 5455000 5460000 5465000 5470000
CD274 (Chr9)
CV−5443
T2−A6X0
P=0.05
P=0.0005
Difference (RPKM)
15
10
5
0
68300000 68500000 68700000 68900000 69100000
RAD51B (Chr14)
P=0.05
P=0.0005
4J−AA1J
C5−A1BE
EA−A50E
EA−A78R
EK−A2H0
EK−A2R8
MY−A5BD
ZJ−A8QQ

Fig. S11b

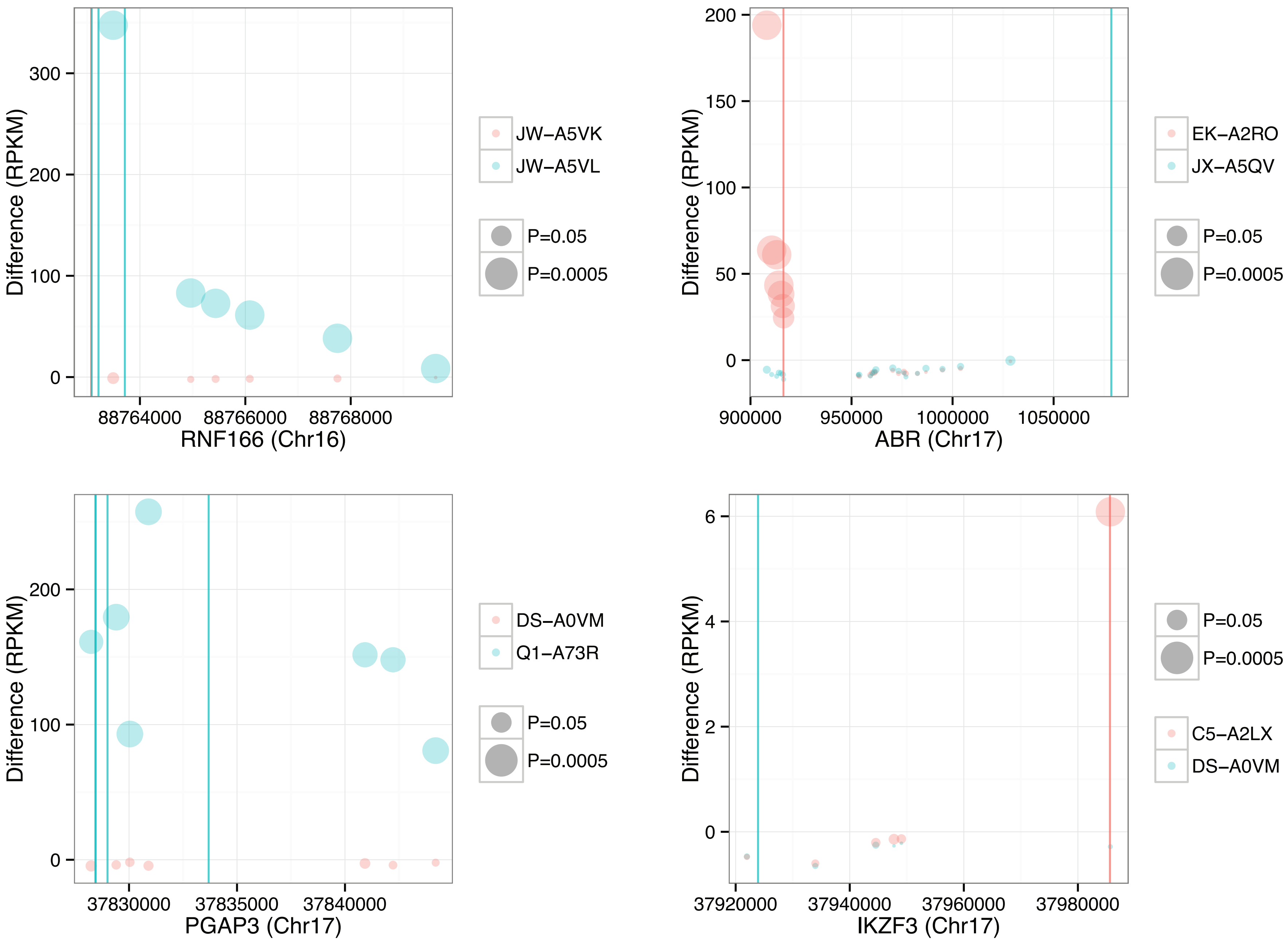
Difference (RPKM)
300
200
100
0
88764000
88766000
88768000
RNF166 (Chr16)
JW−A5VK
JW−A5VL
P=0.05
P=0.0005
200
150
100
50
0
900000
950000
1000000
1050000
ABR (Chr17)
EK−A2RO
JX−A5QV
200
100
0
37830000
37835000
37840000
PGAP3 (Chr17)
DS−A0VM
Q1−A73R
6
4
2
0
37920000
37940000
37960000
37980000
IKZF3 (Chr17)
C5−A2LX
DS−A0VM

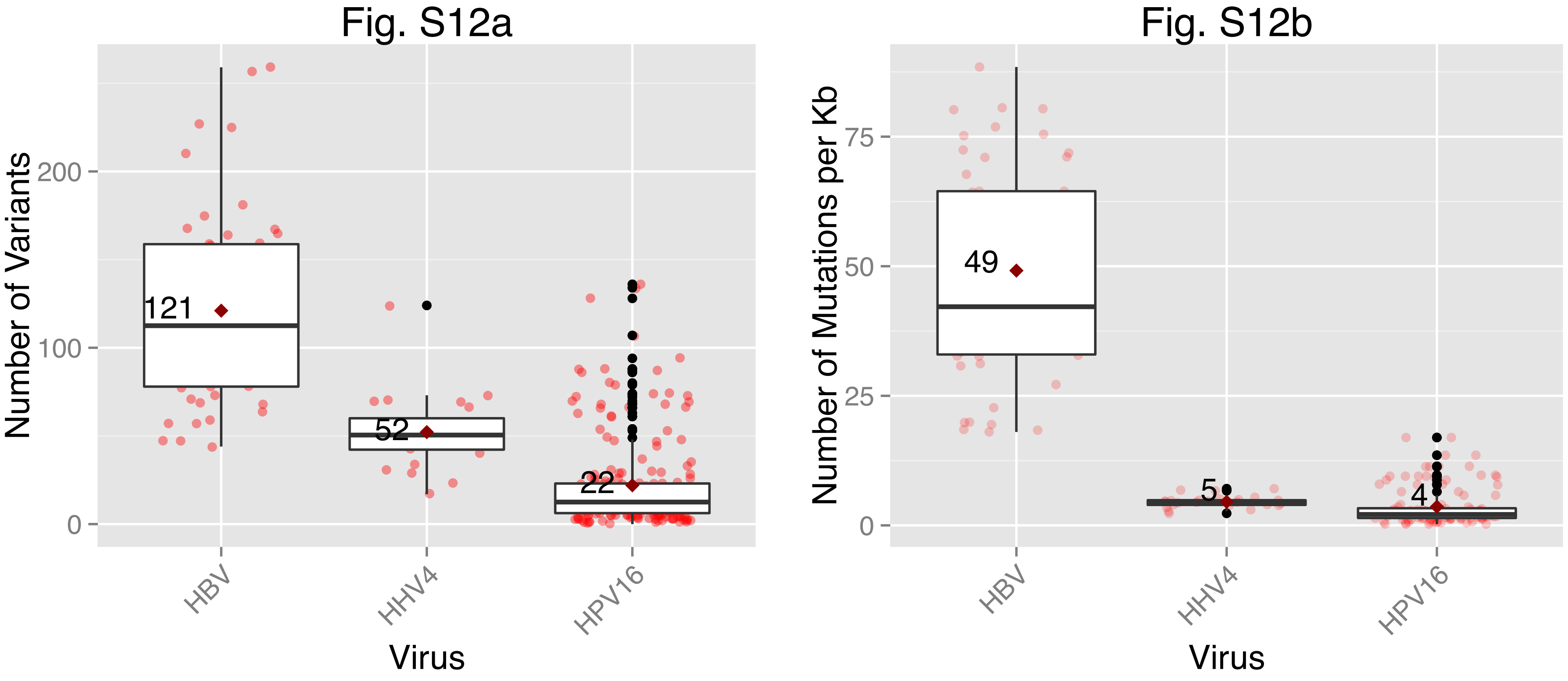
Fig. S12a
Number of Variants
200
100
0
121
52
22
HBV
HHV4
HPV16
Virus
Fig. S12b
Number of Mutations per Kb
75
50
25
0
49
5
4
HBV
HHV4
HPV16
Virus

Fig. S13

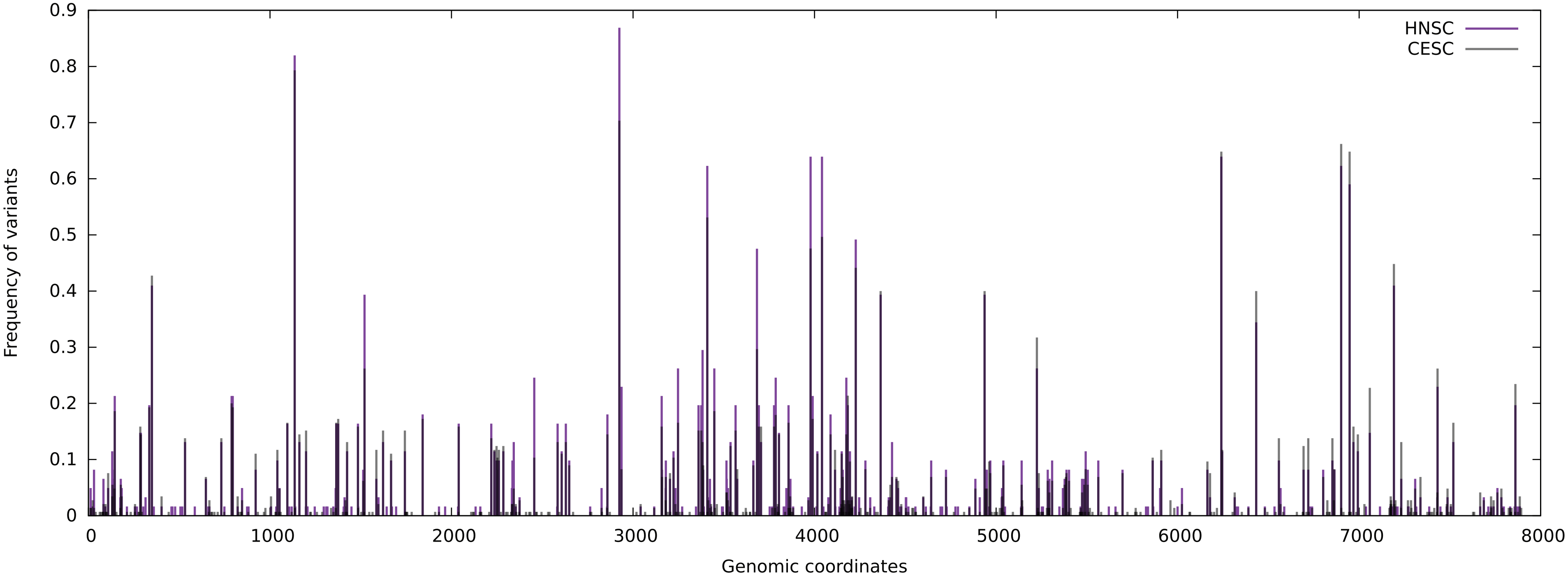

HNSC
CESC
Frequency of variants
Genomic coordinates
0
0.1
0.2
0.3
0.4
0.5
0.6
0.7
0.8
0.9
0
1000
2000
3000
4000
5000
6000
7000
8000

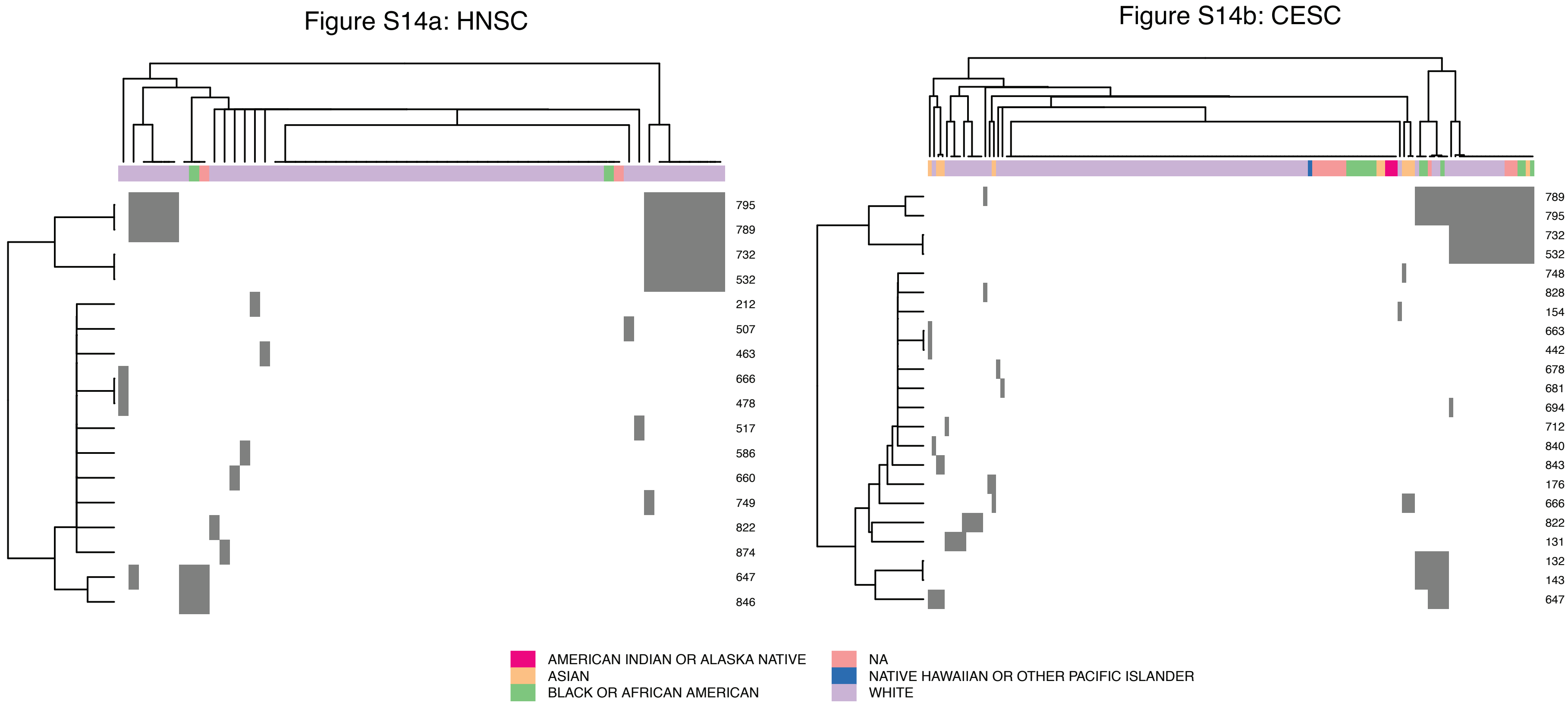
Figure S14a: HNSC
795
789
732
532
212
507
463
666
478
517
586
660
749
822
874
647
846
Figure S14b: CESC
789
795
732
532
748
828
154
663
442
678
681
694
712
840
843
176
666
822
131
132
143
647
AMERICAN INDIAN OR ALASKA NATIVE
ASIAN
BLACK OR AFRICAN AMERICAN
NA
NATIVE HAWAIIAN OR OTHER PACIFIC ISLANDER
WHITE

Table S1: Abbreviations used in the study

a) Abbreviations for 23 cancer types used in the study

**Cancer Types**	**Abbreviations**
Esophageal cancer	ESCA
Stomach adenocarcinoma	STAD
Colon adenocarcinoma	COAD
Rectal adenocarcinoma	READ
Liver hepatocellular carcinoma	LIHC
Pancreatic adenocarcinoma	PAAD
Kidney chromophobe	KICH
Kidney renal clear cell carcinoma	KIRC
Kidney renal papillary cell carcinoma	KIRP
Bladder urothelial carcinoma	BLCA
Prostate adenocarcinoma	PRAD
Uterine carcinosarcoma	UCS
Cervical squamous cell carcinoma and endocervical adenocarcinoma	CESC
Head/neck squamous cell carcinoma	HNSC
Lung squamous cell carcinoma	LUSC
Lung adenocarcinoma	LUAD
Thyroid carcinoma	THCA
Diffuse large B-cell lymphoma	DLBC
Acute myeloid leukemia	LAML
Sarcoma	SARC
Skin cutaneous melanoma	SKCM
Glioblastoma multiforme	GBM
Brain low grade glioma	LGG

b) Abbreviations for viruses used in the study

**Viruses**	**Abbreviations**
Human papillomavirus	HPV
Human hepatitis B and C	HBV and HCV
Human T-cell lymphotropic virus	HTYV
Human herpesvirus	HHV
Kaposi's sarcoma virus	HHV8
Merkel cell polyomavirus	MCV
Human immunodeficiency virus-1	HIV-1
Epstein-Barr virus	EBV or HHV4
BK polyomavirus	BKV
JC polyomavirus	JCV

c) Abbreviations for human genes used in the study

**Genes**	**Abbreviations**
Grainyhead-Like Transcription Factor 2	*GPHL2*
RAD51 Paralog B	*RAD51B*
Cancer Susceptibility Candidate 8	*CASC8*
Cancer Susceptibility Candidate 11	*CASC11*
Post-glycosylphosphatidylinositol attachment to proteins 3	*PGAP3*
Human epidermal growth factor receptor 2	*HER2 or ERBB2*
IKAROS Family Zinc Finger 3	*IKZF3*
Protein Tyrosine Phosphatase, Non-Receptor Type 13	*PTPN13*
Mixed-lineage leukemia 4	*MLL4 or KMT2B*
Myelocytomatosis oncogene cellular homolog	*MYC*
Telomerase Reverse Transcriptase	*TERT*

d) Abbreviations for viral genes used in the study

**Genes**	**Abbreviations**
Epstein-Barr virus (EBV)-encoded RNA 1	*EBER1*

Table S2: The locations of the common integration sites. In column 13, "e" stands for exon and "i" stands for intron.

Sample	Cancer	Virus Type	Start	End	Anchor's strand	Virus Gene	Chr	Start	End	Mate's strand	Host gene	Location	Number of spporting reads pairs
TCGA-FU-A3YQ-01A-11R-A22U-07	CESC	HPV16	4795	5843	-	L1;L2	1	206317539	206318587	+	CTSE	e1;e2;i1;i2	18
TCGA-FU-A3YQ-01A-11R-A22U-07	CESC	HPV16	662	1758	+	E1;E7	1	206317941	206318989	+	CTSE	e2;i1;i2	850
TCGA-FU-A3YQ-01A-11R-A22U-07	CESC	HPV16	66	1162	+	E1;E6;E7	1	206317941	206318989	+	CTSE	e2;i1;i2	754
TCGA-VS-A8QA-01A-11R-A37O-07	CESC	HPV16	241	1337	+	E1;E6;E7	1	206318579	206319627	+	CTSE	e3;i2;i3	26
TCGA-FU-A3YQ-01A-11R-A22U-07	CESC	HPV16	662	1758	+	E1;E7	1	206318676	206319724	+	CTSE	e3;i2;i3	1798
TCGA-FU-A3YQ-01A-11R-A22U-07	CESC	HPV16	662	1758	+	E1;E7	1	206318678	206319726	+	CTSE	e3;i2;i3	803
TCGA-FU-A3YQ-01A-11R-A22U-07	CESC	HPV16	66	1162	+	E1;E6;E7	1	206318678	206319726	+	CTSE	e3;i2;i3	87
TCGA-FU-A3YQ-01A-11R-A22U-07	CESC	HPV16	1967	3063	+	E1;E2	1	206318678	206319726	+	CTSE	e3;i2;i3	33
TCGA-FU-A3YQ-01A-11R-A22U-07	CESC	HPV16	662	1758	+	E1;E7	1	206319756	206320804	+	CTSE	e4;i3;i4	150
TCGA-FU-A3YQ-01A-11R-A22U-07	CESC	HPV16	66	1162	+	E1;E6;E7	1	206319756	206320804	+	CTSE	e4;i3;i4	32
TCGA-FU-A3YQ-01A-11R-A22U-07	CESC	HPV16	662	1758	+	E1;E7	1	206324783	206325831	+	CTSE	e5;i4;i5	45
TCGA-EA-A44S-01A-12R-A26T-07	CESC	HPV16	241	1337	+	E1;E6;E7	13	73987135	73988183	+	LINC00393	3'	92
TCGA-Q1-A5R3-01A-11R-A28H-07	CESC	HPV18	1	689	+	E6;E7	13	73987136	73988184	+	LINC00393	3'	16
TCGA-Q1-A5R3-01A-11R-A28H-07	CESC	HPV18	293	1389	+	E1;E6;E7	13	73987136	73988184	+	LINC00393	3'	2429
TCGA-C5-A8YR-01A-12R-A37O-07	CESC	HPV45	1	618	+	E6;E7	13	73987137	73988185	+	LINC00393	3'	232
TCGA-C5-A8YR-01A-12R-A37O-07	CESC	HPV45	118	1214	+	E1;E6;E7	13	73987137	73988185	+	LINC00393	3'	1532
TCGA-C5-A8YR-01A-12R-A37O-07	CESC	HPV45	714	1810	+	E1;E7	13	73987137	73988185	+	LINC00393	3'	5191
TCGA-JX-A3PZ-01A-11R-A32Y-07	CESC	HPV45	2936	4032	+	E2;E4;E5	13	73987435	73988483	+	LINC00393	3'	1360
TCGA-JX-A3PZ-01A-11R-A32Y-07	CESC	HPV45	290	1386	+	E1;E6;E7	13	73987435	73988483	+	LINC00393	3'	59
TCGA-JX-A3PZ-01A-11R-A32Y-07	CESC	HPV45	2936	4032	+	E2;E4;E5	13	73990121	73991169	+	LINC00393	3'	178
TCGA-JX-A3PZ-01A-11R-A32Y-07	CESC	HPV45	2936	4032	+	E2;E4;E5	13	73990177	73991225	+	LINC00393	3'	174
TCGA-C5-A8YR-01A-12R-A37O-07	CESC	HPV45	1610	2706	+	E1	13	73993882	73994930	+	LINC00393	3'	35
TCGA-EA-A44S-01A-12R-A26T-07	CESC	HPV16	241	1337	+	E1;E6;E7	13	74050705	74051753	+	LINC00393	3'	34
TCGA-EA-A44S-01A-12R-A26T-07	CESC	HPV16	1797	2893	+	E1;E2	13	74071218	74072266	+	LINC00393	3'	28
TCGA-C5-A1BL-01A-11R-A13Y-07	CESC	HPV16	407	1503	+	E1;E6;E7	13	74169189	74170237	+	LINC00393	i1	14
TCGA-C5-A1BL-01A-11R-A13Y-07	CESC	HPV16	407	1503	+	E1;E6;E7	13	74177837	74178885	+	LINC00393	i1	20
TCGA-C5-A1BL-01A-11R-A13Y-07	CESC	HPV16	2191	3239	-	E1;E2	13	74177841	74178889	+	LINC00393	i1	63
TCGA-C5-A1BL-01A-11R-A13Y-07	CESC	HPV16	407	1503	+	E1;E6;E7	13	74187927	74188975	+	LINC00393	i1	21
TCGA-C5-A1BL-01A-11R-A13Y-07	CESC	HPV16	1	681	+	E6;E7	13	74189683	74190731	+	LINC00393	i1	111
TCGA-C5-A1BL-01A-11R-A13Y-07	CESC	HPV16	407	1503	+	E1;E6;E7	13	74189683	74190731	+	LINC00393	i1	33
TCGA-C5-A1BL-01A-11R-A13Y-07	CESC	HPV16	407	1503	+	E1;E6;E7	13	74190249	74191297	+	LINC00393	i1	272
TCGA-C5-A1BL-01A-11R-A13Y-07	CESC	HPV16	407	1503	+	E1;E6;E7	13	74190684	74191732	+	LINC00393	i1	103
TCGA-C5-A1BL-01A-11R-A13Y-07	CESC	HPV16	407	1503	+	E1;E6;E7	13	74190859	74191907	+	LINC00393	i1	42
TCGA-C5-A1BL-01A-11R-A13Y-07	CESC	HPV16	407	1503	+	E1;E6;E7	13	74196062	74197110	+	LINC00393	i1	14
TCGA-C5-A1BL-01A-11R-A13Y-07	CESC	HPV16	407	1503	+	E1;E6;E7	13	74199271	74200319	+	LINC00393	i1	11
TCGA-C5-A1BL-01A-11R-A13Y-07	CESC	HPV16	2992	4088	+	E2;E4;E5	13	74200591	74201639	+	LINC00393	i1	71

TCGA-C5-A1BL-01A-11R-A13Y-07	CESC	HPV16	1	681	+	E6;E7	13	74205394	74206442	+	LINC00393	i1	109
TCGA-C5-A1BL-01A-11R-A13Y-07	CESC	HPV16	2992	4088	+	E2;E4;E5	13	74205394	74206442	+	LINC00393	i1	126
TCGA-C5-A1BL-01A-11R-A13Y-07	CESC	HPV16	3663	4759	+	E2;E5;L2	13	74205394	74206442	+	LINC00393	i1	16
TCGA-C5-A1BL-01A-11R-A13Y-07	CESC	HPV16	407	1503	+	E1;E6;E7	13	74205394	74206442	+	LINC00393	i1	124
TCGA-C5-A1BL-01A-11R-A13Y-07	CESC	HPV16	2191	3239	-	E1;E2	13	74210342	74211390	+	LINC00393	i1	20
TCGA-C5-A1BL-01A-11R-A13Y-07	CESC	HPV16	2191	3239	-	E1;E2	13	74212649	74213697	+	LINC00393	i1	465
TCGA-MY-A5BD-01A-11R-A26T-07	CESC	HPV16	3365	4461	+	E2;E4;E5;L2	14	68616061	68617109	-	RAD51B	i7	71
TCGA-MY-A5BD-01A-11R-A26T-07	CESC	HPV16	3370	4466	+	E2;E4;E5;L2	14	68616471	68617519	+	RAD51B	i7	382
TCGA-EK-A2R8-01A-21R-A18M-07	CESC	HPV45	3469	4517	-	E2;E4;E5;L2	14	68644580	68645628	+	RAD51B	i7	1162
TCGA-EK-A2R8-01A-21R-A18M-07	CESC	HPV45	3469	4517	-	E2;E4;E5;L2	14	68645391	68646439	+	RAD51B	i7	2259
TCGA-EK-A2R8-01A-21R-A18M-07	CESC	HPV45	1	618	+	E6;E7	14	68645393	68646441	+	RAD51B	i7	30
TCGA-EK-A2R8-01A-21R-A18M-07	CESC	HPV45	118	1214	+	E1;E6;E7	14	68645393	68646441	+	RAD51B	i7	287
TCGA-EK-A2R8-01A-21R-A18M-07	CESC	HPV45	714	1810	+	E1;E7	14	68645393	68646441	+	RAD51B	i7	1568
TCGA-EK-A2R8-01A-21R-A18M-07	CESC	HPV45	714	1810	+	E1;E7	14	68650827	68651875	+	RAD51B	i7	76
TCGA-EK-A2R8-01A-21R-A18M-07	CESC	HPV45	2028	3124	+	E1;E2	14	68671205	68672253	+	RAD51B	i7	1756
TCGA-C5-A1BE-01B-11R-A13Y-07	CESC	HPV16	1	684	+	E6;E7	14	68800943	68801991	+	RAD51B	i8	14
TCGA-C5-A1BE-01B-11R-A13Y-07	CESC	HPV16	241	1337	+	E1;E6;E7	14	68800943	68801991	+	RAD51B	i8	1317
TCGA-EA-A78R-01A-11R-A32P-07	CESC	HPV52	243	1339	+	E1;E6;E7	14	68980578	68981626	+	RAD51B	i10	79
TCGA-4J-AA1J-01A-21R-A38B-07	CESC	HPV18	305	1401	+	E1;E6;E7	14	68982642	68983690	+	RAD51B	i10	36
TCGA-4J-AA1J-01A-21R-A38B-07	CESC	HPV45	2219	3267	-	E1;E2	14	68982650	68983698	+	RAD51B	i10	952
TCGA-4J-AA1J-01A-21R-A38B-07	CESC	HPV18	2199	3247	-	E1;E2	14	68982650	68983698	+	RAD51B	i10	6480
TCGA-EA-A78R-01A-11R-A32P-07	CESC	HPV52	243	1339	+	E1;E6;E7	14	69006774	69007822	+	RAD51B	i10	44
TCGA-EA-A78R-01A-11R-A32P-07	CESC	HPV52	243	1339	+	E1;E6;E7	14	69060779	69061827	+	RAD51B	e11;i10	13
TCGA-EA-A50E-01A-21R-A26T-07	CESC	HPV16	238	1334	+	E1;E6;E7	14	69064376	69065424	+	RAD51B	3'	31
TCGA-EA-A50E-01A-21R-A26T-07	CESC	HPV16	238	1334	+	E1;E6;E7	14	69065382	69066430	+	RAD51B	3'	265
TCGA-ZJ-A8QQ-01A-11R-A37O-07	CESC	HPV39	2347	3443	+	E1;E2;E4	14	69148275	69149323	+	RAD51B	3'	18
TCGA-EK-A2H0-01A-11R-A180-07	CESC	HPV39	2347	3443	+	E1;E2;E4	14	69148336	69149384	+	RAD51B	3'	84
TCGA-EK-A2H0-01A-11R-A180-07	CESC	HPV39	145	1241	+	E1;E6;E7	14	69148336	69149384	+	RAD51B	3'	16
TCGA-EK-A2H0-01A-11R-A180-07	CESC	HPV39	145	1241	+	E1;E6;E7	14	69149219	69150267	+	RAD51B	3'	67
TCGA-ZJ-A8QQ-01A-11R-A37O-07	CESC	HPV39	145	1241	+	E1;E6;E7	14	69149416	69150464	+	RAD51B	3'	54
TCGA-JW-A5VK-01A-11R-A28H-07	CESC	HPV16	218	1314	+	E1;E6;E7	16	88762551	88763599	+	RNF166	e6	13
TCGA-JX-A5QV-01A-22R-A28H-07	CESC	HPV16	227	1323	+	E1;E6;E7	16	88762551	88763599	+	RNF166	e6	11
TCGA-JW-A5VL-01A-11R-A28H-07	CESC	HPV16	1	1049	-	E1;E6;E7	16	88762560	88763608	+	RNF166	e6	14
TCGA-JW-A5VL-01A-11R-A28H-07	CESC	HPV16	1	686	+	E6;E7	16	88762689	88763737	+	RNF166	e6	57
TCGA-JW-A5VL-01A-11R-A28H-07	CESC	HPV16	905	2001	+	E1	16	88762689	88763737	+	RNF166	e6	1475
TCGA-JW-A5VL-01A-11R-A28H-07	CESC	HPV16	244	1340	+	E1;E6;E7	16	88762689	88763737	+	RNF166	e6	20711
TCGA-JW-A5VL-01A-11R-A28H-07	CESC	HPV16	1	1049	-	E1;E6;E7	16	88763190	88764238	+	RNF166	e6;i5	558
TCGA-EK-A2RO-01A-11R-A18M-07	CESC	HPV16	1	683	+	E6;E7	17	915768	916816	+	ABR	e17;e18;i16;i17;i18	14

TCGA-EK-A2RO-01A-11R-A18M-07	CESC	HPV16	239	1335	+	E1;E6;E7	17	915768	916816	+	ABR	e17;e18;i16;i17;i18	112
TCGA-JX-A5QV-01A-22R-A28H-07	CESC	HPV16	6045	7093	-	L1	17	1078019	1079067	+	ABR	i1	17
TCGA-Q1-A73R-01A-11R-A33Z-07	CESC	HPV16	5963	7011	-	L1	17	37827920	37828968	+	PGAP3	e7	1264
TCGA-Q1-A73R-01A-11R-A33Z-07	CESC	HPV16	7260	8356	+	N/A	17	37827930	37828978	+	PGAP3	e7	10
TCGA-Q1-A73R-01A-11R-A33Z-07	CESC	HPV16	7260	8356	+	N/A	17	37828480	37829528	+	PGAP3	e6;e7;i5;i6	569
TCGA-Q1-A73R-01A-11R-A33Z-07	CESC	HPV16	5963	7011	-	L1	17	37833163	37834211	+	PGAP3	i3	23
TCGA-DS-A0VM-01A-11R-A10U-07	CESC	HPV16	1763	2859	+	E1;E2	17	37850744	37851792	+	ERBB2,PGAP3	5'	11
TCGA-LP-A5U3-01A-11R-A28H-07	CESC	HPV16	242	1338	+	E1;E6;E7	17	37855194	37856242	+	ERBB2	5'	17
TCGA-DS-A7WF-01A-11R-A352-07	CESC	HPV16	1	597	+	E6;E7	17	37857850	37858898	+	ERBB2	5'	15
TCGA-DS-A7WF-01A-11R-A352-07	CESC	HPV16	6302	7350	-	L1	17	37858203	37859251	+	ERBB2	5'	10
TCGA-LP-A5U3-01A-11R-A28H-07	CESC	HPV16	1	676	+	E6;E7	17	37862784	37863832	+	ERBB2	e1	13
TCGA-LP-A5U3-01A-11R-A28H-07	CESC	HPV16	242	1338	+	E1;E6;E7	17	37862784	37863832	+	ERBB2	e1	1513
TCGA-DS-A0VM-01A-11R-A10U-07	CESC	HPV16	2885	3933	-	E2;E4;E5	17	37862804	37863852	+	ERBB2	e1	15
TCGA-LP-A5U3-01A-11R-A28H-07	CESC	HPV16	242	1338	+	E1;E6;E7	17	37864145	37865193	+	ERBB2	e1;i1	381
TCGA-LP-A5U3-01A-11R-A28H-07	CESC	HPV16	242	1338	+	E1;E6;E7	17	37865045	37866093	+	ERBB2	e2;e3;i1;i2	17
TCGA-C5-A1M9-01A-11R-A13Y-07	CESC	HPV16	246	1342	+	E1;E6;E7	17	37872219	37873267	+	ERBB2	e10;i10	81
TCGA-C5-A1M9-01A-11R-A13Y-07	CESC	HPV16	246	1342	+	E1;E6;E7	17	37873097	37874145	+	ERBB2	e11;i10;i11	582
TCGA-C5-A1M9-01A-11R-A13Y-07	CESC	HPV16	3107	4203	+	E2;E4;E5	17	37873716	37874764	+	ERBB2	e11;i11	72
TCGA-LP-A5U3-01A-11R-A28H-07	CESC	HPV16	242	1338	+	E1;E6;E7	17	37879625	37880673	+	ERBB2	e13;e14;e15;i13;i14;i15	314
TCGA-LP-A5U3-01A-11R-A28H-07	CESC	HPV16	242	1338	+	E1;E6;E7	17	37880518	37881566	+	ERBB2	e16;e17;i15;i16	15
TCGA-LP-A5U3-01A-11R-A28H-07	CESC	HPV16	4845	5893	-	L1;L2	17	37885869	37886917	+	ERBB2	3'	477
TCGA-LP-A5U3-01A-11R-A28H-07	CESC	HPV16	4864	5960	+	L1;L2	17	37886130	37887178	+	ERBB2	3'	12
TCGA-DS-A0VM-01A-11R-A10U-07	CESC	HPV16	6507	7555	-	L1	17	37923397	37924445	+	IKZF3	i7	44
TCGA-C5-A2LX-01A-11R-A18M-07	CESC	HPV16	280	1376	+	E1;E6;E7	17	37985092	37986140	+	IKZF3	e3;i2;i3	14
TCGA-EK-A2PK-01A-11R-A18M-07	CESC	HPV18	289	1385	+	E1;E6;E7	4	87535611	87536659	+	PTPN13	i1	28
TCGA-EK-A2PK-01A-11R-A18M-07	CESC	HPV18	2956	4052	+	E2;E4;E5	4	87535611	87536659	+	PTPN13	i1	518
TCGA-WL-A834-01A-11R-A352-07	CESC	HPV16	2853	3901	-	E2;E4;E5	4	87555884	87556932	+	PTPN13	e2;i1;i2	24
TCGA-WL-A834-01A-11R-A352-07	CESC	HPV16	1	1049	-	E1;E6;E7	4	87555884	87556932	+	PTPN13	e2;i1;i2	44
TCGA-VS-A8QC-01A-11R-A37O-07	CESC	HPV16	241	1337	+	E1;E6;E7	4	87655384	87656432	+	PTPN13	e13;e14;i12;i13;i14	450
TCGA-VS-A8QC-01A-11R-A37O-07	CESC	HPV16	1	1049	-	E1;E6;E7	4	87656077	87657125	+	PTPN13	e15;i14;i15	28
TCGA-VS-A8QC-01A-11R-A37O-07	CESC	HPV16	241	1337	+	E1;E6;E7	4	87656239	87657287	+	PTPN13	e15;i14;i15	11
TCGA-JX-A5QV-01A-22R-A28H-07	CESC	HPV16	6624	7672	-	L1	8	102571740	102572788	+	GRHL2	i4	10
TCGA-IR-A3L7-01A-21R-A213-07	CESC	HPV18	118	1214	+	E1;E6;E7	8	102622681	102623729	+	GRHL2	i8	509
TCGA-IR-A3L7-01A-21R-A213-07	CESC	HPV18	714	1810	+	E1;E7	8	102622681	102623729	+	GRHL2	i8	3407
TCGA-IR-A3L7-01A-21R-A213-07	CESC	HPV18	714	1810	+	E1;E7	8	102623615	102624663	+	GRHL2	i8	18
TCGA-IR-A3L7-01A-21R-A213-07	CESC	HPV18	118	1214	+	E1;E6;E7	8	102624751	102625799	+	GRHL2	i8	13
TCGA-IR-A3L7-01A-21R-A213-07	CESC	HPV18	714	1810	+	E1;E7	8	102624751	102625799	+	GRHL2	i8	128
TCGA-IR-A3L7-01A-21R-A213-07	CESC	HPV18	1558	2654	+	E1	8	102627378	102628426	+	GRHL2	i8	119

TCGA-EK-A2IP-01A-11R-A180-07	CESC	HPV45	622	1718	+	E1;E7	8	128301664	128302712	+	CASC8	e6;i5	161
TCGA-EK-A2IP-01A-11R-A180-07	CESC	HPV45	15	1111	+	E1;E6;E7	8	128301664	128302712	+	CASC8	e6;i5	19
TCGA-FU-A23L-01A-11R-A16R-07	CESC	HPV45	1	618	+	E6;E7	8	128301669	128302717	+	CASC8	e6;i5	77
TCGA-FU-A23L-01A-11R-A16R-07	CESC	HPV45	118	1214	+	E1;E6;E7	8	128301669	128302717	+	CASC8	e6;i5	796
TCGA-FU-A23L-01A-11R-A16R-07	CESC	HPV45	714	1810	+	E1;E7	8	128301669	128302717	+	CASC8	e6;i5	4225
TCGA-FU-A23K-01A-11R-A16R-07	CESC	HPV18	1	688	+	E6;E7	8	128301916	128302964	+	CASC8	e6;i5	145
TCGA-FU-A23K-01A-11R-A16R-07	CESC	HPV18	784	1880	+	E1;E7	8	128301916	128302964	+	CASC8	e6;i5	3201
TCGA-FU-A23K-01A-11R-A16R-07	CESC	HPV18	188	1284	+	E1;E6;E7	8	128301916	128302964	+	CASC8	e6;i5	568
TCGA-FU-A40J-01A-11R-A24H-07	CESC	HPV16	1	687	+	E6;E7	8	128302059	128303107	+	CASC8	e6;i5	732
TCGA-FU-A40J-01A-11R-A24H-07	CESC	HPV16	244	1340	+	E1;E6;E7	8	128302059	128303107	+	CASC8	e6;i5	2277
TCGA-FU-A40J-01A-11R-A24H-07	CESC	HPV16	244	1340	+	E1;E6;E7	8	128305255	128306303	+	CASC8	i5	10
TCGA-FU-A40J-01A-11R-A24H-07	CESC	HPV16	244	1340	+	E1;E6;E7	8	128305374	128306422	+	CASC8	i5	33
TCGA-FU-A40J-01A-11R-A24H-07	CESC	HPV16	244	1340	+	E1;E6;E7	8	128307148	128308196	+	CASC8	i5	26
TCGA-FU-A40J-01A-11R-A24H-07	CESC	HPV16	1733	2829	+	E1;E2	8	128310072	128311120	+	CASC8	i5	11
TCGA-FU-A23L-01A-11R-A16R-07	CESC	HPV45	118	1214	+	E1;E6;E7	8	128311419	128312467	+	CASC8	i5	13
TCGA-FU-A23L-01A-11R-A16R-07	CESC	HPV45	714	1810	+	E1;E7	8	128311419	128312467	+	CASC8	i5	122
TCGA-FU-A40J-01A-11R-A24H-07	CESC	HPV16	244	1340	+	E1;E6;E7	8	128313224	128314272	+	CASC8	i5	15
TCGA-FU-A23L-01A-11R-A16R-07	CESC	HPV45	2576	3672	+	E1;E2;E4	8	128319939	128320987	+	CASC8	i5	53
TCGA-FU-A40J-01A-11R-A24H-07	CESC	HPV16	1	687	+	E6;E7	8	128334850	128335898	+	CASC8	i5	473
TCGA-FU-A40J-01A-11R-A24H-07	CESC	HPV16	244	1340	+	E1;E6;E7	8	128334850	128335898	+	CASC8	i5	531
TCGA-FU-A40J-01A-11R-A24H-07	CESC	HPV16	1	1049	-	E1;E6;E7	8	128334921	128335969	+	CASC8	i5	77
TCGA-FU-A40J-01A-11R-A24H-07	CESC	HPV16	1769	2817	-	E1;E2	8	128334921	128335969	+	CASC8	i5	39
TCGA-FU-A40J-01A-11R-A24H-07	CESC	HPV16	1733	2829	+	E1;E2	8	128337985	128339033	+	CASC8	i5	629
TCGA-EK-A2H1-01A-11R-A180-07	CESC	HPV16	3150	4246	+	E2;E4;E5;L2	8	128400426	128401474	+	CASC8	i5	64
TCGA-EK-A2H1-01A-11R-A180-07	CESC	HPV16	3150	4246	+	E2;E4;E5;L2	8	128403255	128404303	+	CASC8	i5	14
TCGA-EK-A2H1-01A-11R-A180-07	CESC	HPV16	600	1696	+	E1;E7	8	128403255	128404303	+	CASC8	i5	24
TCGA-EK-A2H1-01A-11R-A180-07	CESC	HPV16	1	1085	+	E1;E6;E7	8	128404390	128405438	+	CASC8	i5	89
TCGA-EK-A2H1-01A-11R-A180-07	CESC	HPV16	3150	4246	+	E2;E4;E5;L2	8	128404390	128405438	+	CASC8	i5	69
TCGA-EK-A2H1-01A-11R-A180-07	CESC	HPV16	600	1696	+	E1;E7	8	128404390	128405438	+	CASC8	i5	360
TCGA-EK-A2H1-01A-11R-A180-07	CESC	HPV16	3011	4059	-	E2;E4;E5	8	128404825	128405873	+	CASC8	i5	84
TCGA-UC-A7PF-01A-11R-A352-07	CESC	HPV16	2960	4056	+	E2;E4;E5	8	128432740	128433788	+	CASC8	e5;i4;i5	30
TCGA-UC-A7PF-01A-11R-A352-07	CESC	HPV16	239	1335	+	E1;E6;E7	8	128432740	128433788	+	CASC8	e5;i4;i5	57
TCGA-UC-A7PF-01A-11R-A352-07	CESC	HPV16	2960	4056	+	E2;E4;E5	8	128455327	128456375	+	CASC8	i4	29
TCGA-UC-A7PF-01A-11R-A352-07	CESC	HPV16	2960	4056	+	E2;E4;E5	8	128469703	128470751	+	CASC8	i4	477
TCGA-UC-A7PF-01A-11R-A352-07	CESC	HPV16	239	1335	+	E1;E6;E7	8	128469703	128470751	+	CASC8	i4	10
TCGA-EA-A3HQ-01A-11R-A213-07	CESC	HPV16	1	1049	-	E1;E6;E7	8	128806339	128807387	+	PVT1	5'	21
TCGA-C5-A2M1-01A-11R-A18M-07	CESC	HPV16	588	1684	+	E1;E7	8	128806518	128807566	+	PVT1	5'	18
TCGA-C5-A2M1-01A-11R-A18M-07	CESC	HPV16	3099	4195	+	E2;E4;E5	8	128806518	128807566	+	PVT1	5'	573

TCGA-EA-A3HQ-01A-11R-A213-07	CESC	HPV16	1	1049	-	E1;E6;E7	8	128807651	128808699	+	PVT1	5'	13
TCGA-C5-A2M1-01A-11R-A18M-07	CESC	HPV16	3099	4195	+	E2;E4;E5	8	128807752	128808800	+	PVT1	5'	80
TCGA-LP-A7HU-01A-11R-A33Z-07	CESC	HPV16	1	1048	+	E1;E6;E7	8	128808641	128809689	+	PVT1	5'	33
TCGA-C5-A2M1-01A-11R-A18M-07	CESC	HPV16	588	1684	+	E1;E7	8	128863806	128864854	+	PVT1	5'	26
TCGA-C5-A2M1-01A-11R-A18M-07	CESC	HPV16	3099	4195	+	E2;E4;E5	8	128863806	128864854	+	PVT1	5'	30
TCGA-C5-A3HE-01A-21R-A22U-07	CESC	HPV18	2383	3431	-	E1;E2;E4	8	128865859	128866907	+	PVT1	5'	20
TCGA-C5-A2M1-01A-11R-A18M-07	CESC	HPV16	588	1684	+	E1;E7	8	128866924	128867972	+	PVT1	5'	29
TCGA-C5-A2M1-01A-11R-A18M-07	CESC	HPV16	3099	4195	+	E2;E4;E5	8	128866924	128867972	+	PVT1	5'	12
TCGA-C5-A2M1-01A-11R-A18M-07	CESC	HPV16	3099	4195	+	E2;E4;E5	8	128873074	128874122	+	PVT1	5'	28
TCGA-C5-A3HE-01A-21R-A22U-07	CESC	HPV18	682	1778	+	E1;E7	8	128889878	128890926	+	PVT1	5'	15
TCGA-C5-A3HE-01A-21R-A22U-07	CESC	HPV18	86	1182	+	E1;E6;E7	8	128889878	128890926	+	PVT1	5'	30
TCGA-C5-A2M1-01A-11R-A18M-07	CESC	HPV16	588	1684	+	E1;E7	8	128889887	128890935	+	PVT1	5'	13
TCGA-EA-A3HQ-01A-11R-A213-07	CESC	HPV16	7260	8356	+	N/A	8	128891915	128892963	+	PVT1	5'	35
TCGA-C5-A2M1-01A-11R-A18M-07	CESC	HPV16	588	1684	+	E1;E7	8	128902699	128903747	+	PVT1	e1;i1	158
TCGA-C5-A2M1-01A-11R-A18M-07	CESC	HPV16	1	1028	+	E1;E6;E7	8	128902699	128903747	+	PVT1	e1;i1	12
TCGA-C5-A2M1-01A-11R-A18M-07	CESC	HPV16	3099	4195	+	E2;E4;E5	8	128902699	128903747	+	PVT1	e1;i1	93
TCGA-C5-A1MJ-01A-11R-A14Y-07	CESC	HPV18	612	1660	-	E1;E7	8	128977223	128978271	+	PVT1	i2	182
TCGA-C5-A1MJ-01A-11R-A14Y-07	CESC	HPV18	1478	2574	+	E1	8	128977805	128978853	+	PVT1	i2	235
TCGA-C5-A1M9-01A-11R-A13Y-07	CESC	HPV16	4752	5800	-	L1;L2	8	128989030	128990078	+	PVT1	i2	14
TCGA-C5-A1M9-01A-11R-A13Y-07	CESC	HPV16	3880	4928	-	E5;L2	8	128992750	128993798	+	PVT1	i2	77
TCGA-C5-A1M9-01A-11R-A13Y-07	CESC	HPV16	2480	3576	+	E1;E2;E4	8	128992939	128993987	+	PVT1	i2	21
TCGA-C5-A1M9-01A-11R-A13Y-07	CESC	HPV16	239	1335	+	E1;E6;E7	8	128994095	128995143	-	PVT1	i2	49
TCGA-C5-A1M9-01A-11R-A13Y-07	CESC	HPV16	1	685	+	E6;E7	8	128994230	128995278	+	PVT1	i2	155
TCGA-C5-A1M9-01A-11R-A13Y-07	CESC	HPV16	207	1303	+	E1;E6;E7	8	128994230	128995278	+	PVT1	i2	1418
TCGA-C5-A1M9-01A-11R-A13Y-07	CESC	HPV16	2480	3576	+	E1;E2;E4	8	128994230	128995278	+	PVT1	i2	441
TCGA-C5-A1M9-01A-11R-A13Y-07	CESC	HPV16	803	1899	+	E1;E7	8	128994230	128995278	+	PVT1	i2	359
TCGA-C5-A1M9-01A-11R-A13Y-07	CESC	HPV16	239	1335	+	E1;E6;E7	8	128995642	128996690	-	PVT1	e3;i2;i3	27
TCGA-C5-A1M9-01A-11R-A13Y-07	CESC	HPV16	1	1049	-	E1;E6;E7	8	128995808	128996856	+	PVT1	e3;i2;i3	21
TCGA-C5-A1M9-01A-11R-A13Y-07	CESC	HPV16	1	685	+	E6;E7	8	128996037	128997085	+	PVT1	e3;i2;i3	117
TCGA-C5-A1M9-01A-11R-A13Y-07	CESC	HPV16	207	1303	+	E1;E6;E7	8	128996037	128997085	+	PVT1	e3;i2;i3	529
TCGA-C5-A1M9-01A-11R-A13Y-07	CESC	HPV16	803	1899	+	E1;E7	8	128996037	128997085	+	PVT1	e3;i2;i3	199
TCGA-C5-A1M9-01A-11R-A13Y-07	CESC	HPV16	1	1049	-	E1;E6;E7	8	129000896	129001944	+	PVT1	e4;i3;i4	291
TCGA-C5-A1M9-01A-11R-A13Y-07	CESC	HPV16	4752	5800	-	L1;L2	8	129000896	129001944	+	PVT1	e4;i3;i4	17
TCGA-C5-A1M9-01A-11R-A13Y-07	CESC	HPV16	1	685	+	E6;E7	8	129001243	129002291	+	PVT1	e4;i3;i4	459
TCGA-C5-A1M9-01A-11R-A13Y-07	CESC	HPV16	207	1303	+	E1;E6;E7	8	129001243	129002291	+	PVT1	e4;i3;i4	1964
TCGA-C5-A1M9-01A-11R-A13Y-07	CESC	HPV16	803	1899	+	E1;E7	8	129001243	129002291	+	PVT1	e4;i3;i4	588
TCGA-C5-A1M9-01A-11R-A13Y-07	CESC	HPV16	1	1049	-	E1;E6;E7	8	129004909	129005957	+	PVT1	i4	11
TCGA-C5-A1M9-01A-11R-A13Y-07	CESC	HPV16	1	685	+	E6;E7	8	129006811	129007859	+	PVT1	i4	35

TCGA-C5-A1M9-01A-11R-A13Y-07	CESC	HPV16	207	1303	+	E1;E6;E7	8	129006811	129007859	+	PVT1	i4	75
TCGA-C5-A1M9-01A-11R-A13Y-07	CESC	HPV16	803	1899	+	E1;E7	8	129006811	129007859	+	PVT1	i4	40
TCGA-C5-A1M9-01A-11R-A13Y-07	CESC	HPV16	1	685	+	E6;E7	8	129008846	129009894	+	PVT1	i4	52
TCGA-C5-A1M9-01A-11R-A13Y-07	CESC	HPV16	207	1303	+	E1;E6;E7	8	129008846	129009894	+	PVT1	i4	155
TCGA-C5-A1M9-01A-11R-A13Y-07	CESC	HPV16	803	1899	+	E1;E7	8	129008846	129009894	+	PVT1	i4	73
TCGA-C5-A1M9-01A-11R-A13Y-07	CESC	HPV16	1	1049	-	E1;E6;E7	8	129009965	129011013	+	PVT1	i4	15
TCGA-C5-A1M9-01A-11R-A13Y-07	CESC	HPV16	1	1049	-	E1;E6;E7	8	129013258	129014306	+	PVT1	i4	57
TCGA-C5-A1M9-01A-11R-A13Y-07	CESC	HPV16	6308	7356	-	L1	8	129013258	129014306	+	PVT1	i4	145
TCGA-C5-A1M9-01A-11R-A13Y-07	CESC	HPV16	207	1303	+	E1;E6;E7	8	129014350	129015398	+	PVT1	i4	12
TCGA-C5-A1M9-01A-11R-A13Y-07	CESC	HPV16	1	685	+	E6;E7	8	129021317	129022365	+	PVT1	i4	10
TCGA-C5-A1M9-01A-11R-A13Y-07	CESC	HPV16	207	1303	+	E1;E6;E7	8	129021317	129022365	+	PVT1	i4	10
TCGA-C5-A1M9-01A-11R-A13Y-07	CESC	HPV16	4752	5800	-	L1;L2	8	129022050	129023098	+	PVT1	i4	27
TCGA-C5-A1M9-01A-11R-A13Y-07	CESC	HPV16	207	1303	+	E1;E6;E7	8	129022425	129023473	+	PVT1	i4	23
TCGA-BA-4077-01B-01R-1436-07	HNSC	HPV16	279	1375	+	E1;E6;E7	14	68699742	68700790	+	RAD51B	i7	539
TCGA-BA-4077-01B-01R-1436-07	HNSC	HPV16	279	1375	+	E1;E6;E7	14	68701191	68702239	+	RAD51B	i7	590
TCGA-BA-4077-01B-01R-1436-07	HNSC	HPV16	1	685	+	E6;E7	14	68703642	68704690	+	RAD51B	i7	47
TCGA-BA-4077-01B-01R-1436-07	HNSC	HPV16	279	1375	+	E1;E6;E7	14	68703642	68704690	+	RAD51B	i7	831
TCGA-BA-4077-01B-01R-1436-07	HNSC	HPV16	279	1375	+	E1;E6;E7	14	68706373	68707421	+	RAD51B	i7	48
TCGA-BA-4077-01B-01R-1436-07	HNSC	HPV16	279	1375	+	E1;E6;E7	14	68738593	68739641	+	RAD51B	i7	15
TCGA-BA-4077-01B-01R-1436-07	HNSC	HPV16	279	1375	+	E1;E6;E7	14	68741043	68742091	+	RAD51B	i7	14
TCGA-BA-4077-01B-01R-1436-07	HNSC	HPV16	2932	3980	-	E2;E4;E5	14	68741058	68742106	+	RAD51B	i7	73
TCGA-BA-4077-01B-01R-1436-07	HNSC	HPV16	1	685	+	E6;E7	14	68758058	68759106	+	RAD51B	e8;i7;i8	33
TCGA-BA-4077-01B-01R-1436-07	HNSC	HPV16	279	1375	+	E1;E6;E7	14	68758058	68759106	+	RAD51B	e8;i7;i8	119
TCGA-BA-4077-01B-01R-1436-07	HNSC	HPV16	1	685	+	E6;E7	14	68877658	68878706	+	RAD51B	e9;i8;i9	39
TCGA-BA-4077-01B-01R-1436-07	HNSC	HPV16	279	1375	+	E1;E6;E7	14	68877658	68878706	+	RAD51B	e9;i8;i9	67
TCGA-CN-A6V7-01A-12R-A34R-07	HNSC	HPV16	2851	3947	+	E2;E4;E5	14	68913189	68914237	+	RAD51B	i9	228
TCGA-CN-A6V7-01A-12R-A34R-07	HNSC	HPV16	241	1337	+	E1;E6;E7	14	68913189	68914237	+	RAD51B	i9	29
TCGA-CN-A6V7-01A-12R-A34R-07	HNSC	HPV16	2851	3947	+	E2;E4;E5	14	68914063	68915111	+	RAD51B	i9	41
TCGA-BA-4077-01B-01R-1436-07	HNSC	HPV16	1	685	+	E6;E7	14	68963654	68964702	+	RAD51B	i10	10
TCGA-BA-4077-01B-01R-1436-07	HNSC	HPV16	279	1375	+	E1;E6;E7	14	68963654	68964702	+	RAD51B	i10	17
TCGA-T2-A6X0-01A-11R-A34R-07	HNSC	HPV16	238	1334	+	E1;E6;E7	9	5449916	5450964	+	CD274	e1;i1	56
TCGA-CV-5443-01A-01R-1514-07	HNSC	HPV16	6255	7303	-	L1	9	5464248	5465296	+	CD274	i4	17
TCGA-DD-A119-01A-11R-A131-07	LIHC	HBV	1363	2413	-	C;Polymerase;X	19	36212079	36213129	+	KMT2B	e3;i3	30
TCGA-DD-A3A3-01A-11R-A22L-07	LIHC	HBV	1	1049	-	Polymerase;S	19	36212341	36213389	+	KMT2B	e3;e4;i3;i4	58
TCGA-UB-A7ME-01A-11R-A33J-07	LIHC	HBV	1154	2250	+	C;Polymerase;X	19	36212883	36213931	+	KMT2B	e4;e5;e6;i3;i4;i5	10
TCGA-CC-A9FS-01A-11R-A37K-07	LIHC	HBV	1	1049	-	Polymerase;S	19	36212974	36214022	+	KMT2B	e4;e5;e6;i3;i4;i5	11
TCGA-DD-A116-01A-11R-A131-07	LIHC	HBV	1	1051	-	Polymerase;S	19	36213523	36214573	+	KMT2B	e5;e6;e7;i5;i6;i7	17
TCGA-ED-A8O6-01A-11R-A36F-07	LIHC	HBV	180	1228	-	Polymerase;S	19	36213760	36214808	+	KMT2B	e6;e7;e8;i5;i6;i7	23

TCGA-CC-A9FS-01A-11R-A37K-07	LIHC	HBV	1167	2263	+	C;Polymerase;X	19	36213808	36214856	+	KMT2B	e6;e7;e8;i5;i6;i7	25
TCGA-CC-5258-01A-01R-A131-07	LIHC	HBV	1178	2278	+	C;Polymerase;X	19	36214156	36215206	+	KMT2B	e6;e7;e8;i6;i7;i8	16
TCGA-CC-5258-01A-01R-A131-07	LIHC	HBV	1178	2278	+	C;Polymerase;X	19	36214260	36215310	+	KMT2B	e7;e8;i6;i7;i8	40
TCGA-CC-A3MB-01A-11R-A213-07	LIHC	HBV	1098	2194	+	C;Polymerase;X	5	1270683	1271731	+	TERT	e8;i7;i8	26
TCGA-CC-A3MB-01A-11R-A213-07	LIHC	HBV	130	1226	+	Polymerase;S	5	1272251	1273299	+	TERT	e7;i6;i7	14
TCGA-CC-A3MB-01A-11R-A213-07	LIHC	HBV	1098	2194	+	C;Polymerase;X	5	1272251	1273299	+	TERT	e7;i6;i7	31
TCGA-CC-A3MB-01A-11R-A213-07	LIHC	HBV	1098	2194	+	C;Polymerase;X	5	1273201	1274249	+	TERT	i6	45
TCGA-CC-A3MB-01A-11R-A213-07	LIHC	HBV	1794	2890	+	C;X	5	1273247	1274295	+	TERT	i6	10
TCGA-CC-A3MB-01A-11R-A213-07	LIHC	HBV	1098	2194	+	C;Polymerase;X	5	1273247	1274295	+	TERT	i6	65
TCGA-G3-A25U-01A-11R-A16W-07	LIHC	HBV	1	1049	-	Polymerase;S	5	1294138	1295186	+	TERT	e1;e2;i1	21
